# Enzymatic tools for the treatment of caries-associated biofilms: Investigating the enzymes of a putative polysaccharide utilization locus from *Prevotella melaninogenica* and their application against *Streptococcus mutans* biofilm

**DOI:** 10.1007/s11274-026-05104-8

**Published:** 2026-07-29

**Authors:** Dylan James Erasmus, Adelita Carolina Santiago, Sebastião Pratavieira, Neil Thomas Stacey, Igor Polikarpov

**Affiliations:** 1 São Carlos Institute of Physics, University of São Paulo, Avenida Trabalhador São-carlense, 400, Parque Arnold Schimidt, São Carlos, 13566-590 SP Brazil; 2 University of the Witwatersrand, Johannesburg (Wits), 1 Jan Smuts Avenue, Braamfontein, Johannesburg, 2001 Gauteng South Africa

**Keywords:** Biofilm degradation, Caries, *Streptococcus mutans*, Polysaccharide utilization locus (PUL), *Prevotella melaninogenica*

## Abstract

We identified a potential cluster of genes in the organism *Prevotella melaninogenica* which was predicted to represent a polysaccharide utilization locus (PUL). Based on the knowledge that PUL glycoside hydrolase (GH) enzymes are frequently co-expressed to saccharify a complex polysaccharide, and that one of the enzymes (PmGH87) is known to hydrolyse a major linkage of the extracellular polymeric substance (EPS) of cariogenic biofilms, we hypothesized that the predicted PUL (pPmPUL) GHs may be relevant to the degradation of biofilms associated with oral disease. To test this, the pPmPUL GHs were selected for cloning, recombinant expression and further study. GHs—consisting of a family 87 mutanase (PmGH87), a family 97 glucosidase (PmGH97) and an enzyme of unknown function assigned to the GH71/99 superfamily (PmGH99)—were characterised both structurally and biochemically and were assessed for their ability to degrade caries-associated *Streptococcus mutans* biofilms formed in vitro, both individually and in combinations. The results were compared to a previously described heterologous combination of complex carbohydrate active enzymes (PmGH87 and CoGH66). Both assays quantifying the relative change in biomass and confocal microscope images confirm the ability of pPmPUL GHs to remove *S. mutans* biofilm.

**Supplementary Information:**

The online version contains supplementary material available at 10.1007/s11274-026-05104-8.

## Introduction

Oral diseases such as dental caries and periodontitis represent a significant healthcare challenge, encompassing adverse individual health outcomes and reduced quality of life, together with substantial societal and economic burdens, even in developed countries (Janakiram and Dye 2020, Marcenes et al. 2013). Moreover, it has been shown to negatively impact systemic health (Tattar et al. 2025, Genco and Sanz 2020, Jin et al. 2016). 

The human oral microbiome comprises highly complex and diverse microbial communities, occupying distinct ecological niches within saliva and biofilms attached to both soft and hard oral tissues. Its composition varies between individuals and transiently in response to changing environmental conditions. Collectively, these communities may encompass upwards of 600 species inhabiting the oral cavity (Dewhirst et al. 2010). 

Biofilms that form on tooth surfaces are referred to as dental plaque and are associated with oral diseases such as caries and periodontitis (Janakiram and Dye 2020, Bowen et al. 2018). In particular, the formation of carious lesions is driven by the progressive demineralization of enamel due to sustained exposure to pH values below the critical threshold of (~ 5.5) (Janus et al. 2016). 

There is no single pathogenic agent responsible for caries development. Instead, it is increasingly viewed as the result of a dysbiotic state driven by deleterious shifts in microbial populations, with an overrepresentation of harmful microorganisms (MOs) that produce extracellular polymeric substances (EPS), and acidic metabolites in response to the ingestion of sugars and simple carbohydrates (Colombo and Tanner 2019). 

However, among these MOs, mutans-type oral streptococci (*Mutans streptococci*; MS) are particularly notable. They have been isolated from cariogenic lesions (Colombo and Tanner 2019), act as principal EPS producers (Klein et al. 2015), dominate in sucrose-rich environments (Ren et al. 2019, Nielsen et al. 2024), and their metabolism of sugars and production of dense biofilm matrix create localized acidic microenvironments (Colombo and Tanner 2019, Hanada 2026). Clinical strains of *S. mutans* often possess several virulence factors that enhance their ability to adhere to the tooth surface and to produce and tolerate acid. Additionally, they are particularly prolific biofilm producers, consequently, they can act as keystone species in the pathogenesis of dental caries, even when representing a relatively minor fraction of the population (Klein et al. 2015, Hanada 2026). 

The biofilm matrix produced by MS is largely comprised of glucans. These homopolymers of glucose are produced extracellularly by the action of glucosyltransferase on sucrose—although they have been shown to accept other oligosaccharides as substrates (Bowen and Koo 2011) —they comprise α-(1→3)-, α-(1→6)-, and, to a lesser extent, α-(1→4)-linkages (Klein et al. 2015; Bowen and Koo 2011; Ernst et al. 2024). These glucans are also a significant component of pooled dental plaque formed in situ (Bowen and Koo 2011). Other important matrix components of dental plaque include proteins of both host and bacterial origin (Bowen and Koo 2011) and extracellular DNA (eDNA) (Klein et al. 2015). Some oral MOs including *Candida albicans* and *Porphyromonas gingivalis* produce biofilms which contain polysaccharides comprising mannose residues with α-(1→6)-linked main chains and α-(1→2)-linked side chains (Pierce et al. 2017; Rangarajan et al. 2013). 

*P. gingivalis* is a bacterium recognized as a keystone organism in the pathogenesis of periodontitis (Hajishengallis et al. 2012; Curtis et al. 2025). The yeast *C. albicans* is normally a commensal member of the oral microbiome, however, under certain conditions, it can function as an opportunistic pathogen. It is known to colonize denture surfaces and is a major etiological agent of denture stomatitis, a form of oral candidiasis. Oral candidiasis occurs particularly in children, individuals with immunosuppression and may also arise as a side effect of certain medications. *C. albicans* has also been isolated from caries, within mixed kingdom biofilms, together with bacteria, where it may be implicated in disease pathogenesis by enhancing EPS production, providing a physical scaffold for oral bacteria to adhere to (Janus et al. 2016; Koo and Bowen 2014), and increasing resistance to antimicrobials (Janus et al. 2016; Pierce et al. 2017). On the other hand, it may also play the role of a commensal through the following mechanisms: allowing healthy recolonization of the oral cavity after antimicrobial challenge, providing a niche for other strictly anaerobic commensal MOs, directly raising the pH of the extracellular milieu in response to acidification and decreasing the virulence of pathogenic MOs (Janus et al. 2016), which is significant for caries pathogenesis.

Modern preventive strategies against oral disease employ a three-pronged approach involving: mechanical removal of the biofilm matrix, regular exposure to low concentrations of fluoride to strengthen enamel and prevent demineralization, and the application of antimicrobial agents (Janakiram and Dye 2020; Zilm et al. 2022). However, it is important to consider the limitations of these strategies.

Orthodontic appliances can complicate oral hygiene by limiting or hindering access to the biofilm during mechanical cleaning, often leading to localized enamel demineralization and the formation of characteristic white spot lesions. Exposure to fluoride does not directly address the issues of dysbiosis nor caries-associated biofilm. Antimicrobial agents act indiscriminately, disrupting the oral cavity’s eubiosis, potentially exacerbating disease (Janus et al. 2016), and can result in undesirable side effects (Rajendiran et al. 2021; Poppolo Deus and Ouanounou 2022). Additionally, the efficacy of antimicrobials may be reduced by limited penetration through the biofilm matrix (von Ohle et al. 2010; Ren et al. 2019; Cardoso et al. 2011; Koo et al. 2017). 

The inclusion of enzyme-based treatments in dental hygiene strategies therefore represents an attractive concept. Enzymes act directly on the biofilm matrix, complementing existing mechanical and antimicrobial approaches without disrupting the delicate microbial ecology. Additionally, they offer a gentler and more specific treatment than chemical antimicrobials, potentially reducing the occurrence of undesirable side effects and preserving healthy oral ecology. Furthermore, enzymes are intrinsically biodegradable, making them potentially environmentally friendly alternatives in oral care. In vitro studies employing endo-α-(1→3)-glucanase (mutanase; EC: 3.2.1.59) and endo-α-(1→6)-glucanase (endo-dextranase; EC: 3.2.1.11) enzymes to combat oral biofilms have been performed, and combinations of the two have been shown to work synergistically to enhance biofilm removal (Nielsen et al. 2024; Del Rey et al. 2024; Cortez et al. 2023; Pleszczyńska et al. 2017; Pozelli Macedo et al. 2024; Macedo et al. 2026). Other combinations of enzymes have been tested against biofilms grown both in vitro and in situ with varying degrees of success (Nielsen et al. 2024; Macedo et al. 2026). 

Members of the phylum *Bacteroidota* possess polysaccharide utilization loci (PUL; singular: locus), discrete clusters of co-expressed genes specialized for the degradation of a specific and complex glycan (Grondin et al. 2017; McKee et al. 2021; Martens et al. 2009). PUL frequently consist of gene regulators, transporters, carbohydrate binding proteins, and carbohydrate active enzymes (CAZymes) (Martens et al. 2009). For more information on the discovery and nomenclature of PUL the reader is referred to the Sus (Smith and Salyers 1991; Reeves et al. 1996, 1997; D’Elia and Salyers 1996) and Sus-like paradigm (Martens et al. 2009). The presence, gene composition, and substrate specificity of a PUL can be determined experimentally by quantifying changes in normalized transcription of the genes—or presence of the proteins they encode—when the organism is grown on different glycan substrates in comparison to a control (Reeves et al. 1997; Cuskin et al. 2015; Feng et al. 2022; Nakamura et al. 2023; Murovec and Accetto 2024). 

Recently, a bioinformatics approach was developed for the prediction of PUL in *Bacteroidota* species (Terrapon et al. 2015, 2018), identifying genomic regions likely to represent a PUL based on gene proximity and the presence of a conserved SusC/SusD-like gene pair involved in glycan binding and uptake. This approach has been used to predict 81,235 distinct PULs in 2,494 *Bacteroidetes* species, which can be accessed via (CAZy: PULDB, Polysaccharide-Utilization Loci DataBase). The tool was benchmarked against two databases of experimentally determined PULs (each representing a distinct microorganism: *Bacteroides thetaiotaomicron* and *Bacteroides ovatus*, respectively), achieving > 86% accurate recall of PUL genes with < 8% erroneous additions (Terrapon et al. 2015). It was further tested against *Flavobacterium johnsoniae*—a species distant from the two used to calibrate the prediction tool—to confirm > 80% retrieval of PUL (Lapébie et al. 2019). However, it is currently limited in its ability to correctly delineate the boundaries between adjacent PUL as well as the recognition of non-contiguous gene pairs belonging to the same PUL.

Recently Cortez and collaborators successfully expressed two novel recombinant GHs, including a GH87 mutanase from *Prevotella melaninogenica* (PmGH87), and evaluated their ability to degrade *S. mutans* biofilm (Cortez et al. 2023). Studies with PmGH87 in combination with various GH66 endo-dextranases demonstrate that the presence of PmGH87 was necessary for a significant biofilm removal effect and that the inclusion of enzymes from other families may exhibit synergistic effects enhancing overall biofilm degradation (Cortez et al. 2023; Pozelli Macedo et al. 2024; Macedo et al. 2026). 

Further bioinformatics analysis using the PULdb tool reveals that PmGH87 may belong to a putative PUL. In this paper, the authors endeavour to describe the novel putative *P. melaninogenica* PUL (pPmPUL), to explore—biochemically and structurally—the GHs therein, and to present and validate the hypothesis that combinations of these GHs may yield synergistic effects in the degradation of biofilms formed by clinically relevant oral bacteria.

## Materials and methods

### Bioinformatic analysis and target selection

The predicted PUL (pPmPUL) was identified in *P. melaninogenica* using the CAZy PUL database (PULDB; http://www.cazy.org/PULDB/), by searching for genes homologous to (PmGH87; GenBank: WP_013264413.1) sourced from *P. melaninogenica DSM 7089* (Cortez et al. 2023). As *P. melaninogenica DSM 7089* was not present in PULDB, *P. melaninogenica D18* (PUL 5) was used as a reference, and homologous proteins in DSM 7089 were identified using protein BLAST (Table S1).

Signal peptide predictions were performed using SignalP 6.0 (Almagro Armenteros et al. 2019), and subcellular localization was predicted using DeepLoc-Pro (Moreno et al. 2024). Sequence alignments and phylogenetic analyses were conducted using CLUSTALW and ESPript3, respectively. *P. melaninogenica* (DSM 7089/ATCC 25845; NCBI: txid553174) predicted PUL (pPmPUL) GHs comprise: (PmGH87; GenBank ID: WP_013264413.1), (PmGH99; GenBank ID: WP_013264030.1) and (PmGH97; GenBank ID: WP_013264838.1). Protein structures were built with AlphaFold (Jumper et al. 2021), visualisation and alignments were performed using PyMOL.

### Enzyme source, cloning and purification

Genes encoding novel target enzymes (PmGH97 and PmGH99) were amplified from genomic DNA and cloned into the pETTRXA-1a/LIC expression vector as previously described (Camilo and Polikarpov 2014) (see Supplementary Materials for details). Recombinant proteins were expressed in *Escherichia coli* systems (DH5-α for plasmid cloning; Rosetta (DE3) or ArcticExpress (DE3) for expression) and purified using Ni²⁺ affinity chromatography followed by tag removal via TEV protease digestion and a second affinity purification step (see Supplementary Materials for details).

Protein purity was assessed by SDS-PAGE, and concentrations were determined spectrophotometrically at 280 nm using calculated extinction coefficients. Unless otherwise stated, enzymes were stored at 2 mg/ml in a Tris–NaCl storage buffer (see Supplementary Materials for more details).

Transformed cells expressing recombinant PmGH87 and CoGH66 were obtained previously and the enzymes were expressed and purified following established protocols (Cortez et al. 2023).

### Biofilm-forming bacterial cultures

Glycerinated stock cultures of *Streptococcus mutans* UA159 maintained at -80 °C were reactivated on BHI agar plates for 48 h at (37 °C; 10% CO₂) prior to the preparation of preinocula to be used in the production of biofilm, either to be lyophilized for use as an enzymatic substrate or in microtiter plates for biofilm assays. (as described in Supplementary Materials)

Prior to biofilm assays, overnight cultures (preinocula) of *S. mutans* in tryptone–yeast extract broth (TYEB) supplemented with glucose were standardized spectrophotometrically to an optical density of 1.0 ± 0.05, diluted 1:100 in fresh TYEB supplemented with sucrose (1% w/v) and grown in standard 24/96-well microtiter plates for 24 h at (37 °C; 10% CO₂) (see Supplementary Materials for details) prior to application of enzymatic treatments.

### Biofilm substrate and glucan preparation

As commercial substrates for mutanases are not available, lyophilized *S. mutans* ‘whole biofilm’ (WBF) pellets were prepared, following previously described protocols (Spinola et al. 2019; Cortez et al. 2023).

Water-soluble (WSG) and water-insoluble glucan (WIG) extracts were prepared from WBF via sequential sonication, centrifugation, ethanol precipitation, and washing steps. Final preparations were resuspended in Milli-Q water and lyophilized prior to use. (see Supplementary Materials for details).

### Enzymatic assays

Enzymatic activities were determined colorimetrically by quantifying the amount of product released using standard curves. Unless otherwise stated, activity was determined using the dinitrosalicylic acid (DNS) method to quantify reducing sugars (Miller 1959). For reactions involving pNP-α-D-glucopyranoside (pNP-α-Glc), activity was measured via absorbance at 405 nm following alkaline quenching.

Initial substrate screening was performed against polysaccharide substrates (soluble starch, lyophilized *S. mutans* biofilm (WBF) and commercial *Leuconostoc* dextran). The pH profiles of PmGH97 and PmGH99 were determined against pNP-α-Glc and WBF, respectively, using appropriate reaction conditions as described (Supplementary Materials).

The activity of pPmPUL enzymes (PmGH87, PmGH97 and PmGH99) was assessed against various substrates prepared from *S. mutans* biofilm (WSG, WIG and WBF) by quantifying the amount of glucose equivalents released as a function of time (10–30 min).

Additional assays were performed to evaluate PmGH99 activity against yeast-derived substrates using whole *Saccharomyces cerevisiae* cells. Reaction conditions and incubation times were varied as indicated, and activity was quantified using the DNS method.

### Biofilm degradation assays

Biofilm degradation was quantified relative to controls, using crystal violet staining as a proxy for total biomass, and was performed as follows: *S. mutans* biofilms were formed for 24 h in 96-well plates as described previously. Following biofilm formation, non-adherent cells were removed by washing with saline solution. Subsequently, enzymatic treatments were applied at varying concentrations for 4 h. followed by washing, staining and solubilization steps. The same steps at the absence of enzymes were used in control experiments. Absorbance was measured at 570 nm, and results were expressed as % biofilm removal relative to untreated controls.

Enzymatic treatments involved: varying concentrations of pPmPUL enzymes (PmGH87, PmGH97 and PmGH99) and CoGH66 applied individually, binary combinations (total dose fixed at 1 mg/ml) of either PmGH87 and PmGH97 or PmGH87 and CoGH66 (positive control), or ternary combinations of pPmPUL enzymes (total dose fixed at 1 mg/ml).

### Confocal laser scanning fluorescence microscopy

Biofilm architecture following enzymatic treatment was assessed via confocal laser scanning fluorescence microscopy (CLSM; Zeiss LSM 780), utilizing endogenous fluorescence of the biofilms, and with appropriate excitation sources and detection settings. Biofilms were prepared in 24-well microtiter plates under standardized conditions, treated with enzyme solutions for 2 h, and washed with saline prior to imaging (see Supplementary Materials for details and validation of experimental conditions).

All experiments were performed under standardized conditions with appropriate controls (see Supplementary Materials for details).

## Results

### PUL organisation and protein localisation

**Fig. 1:**
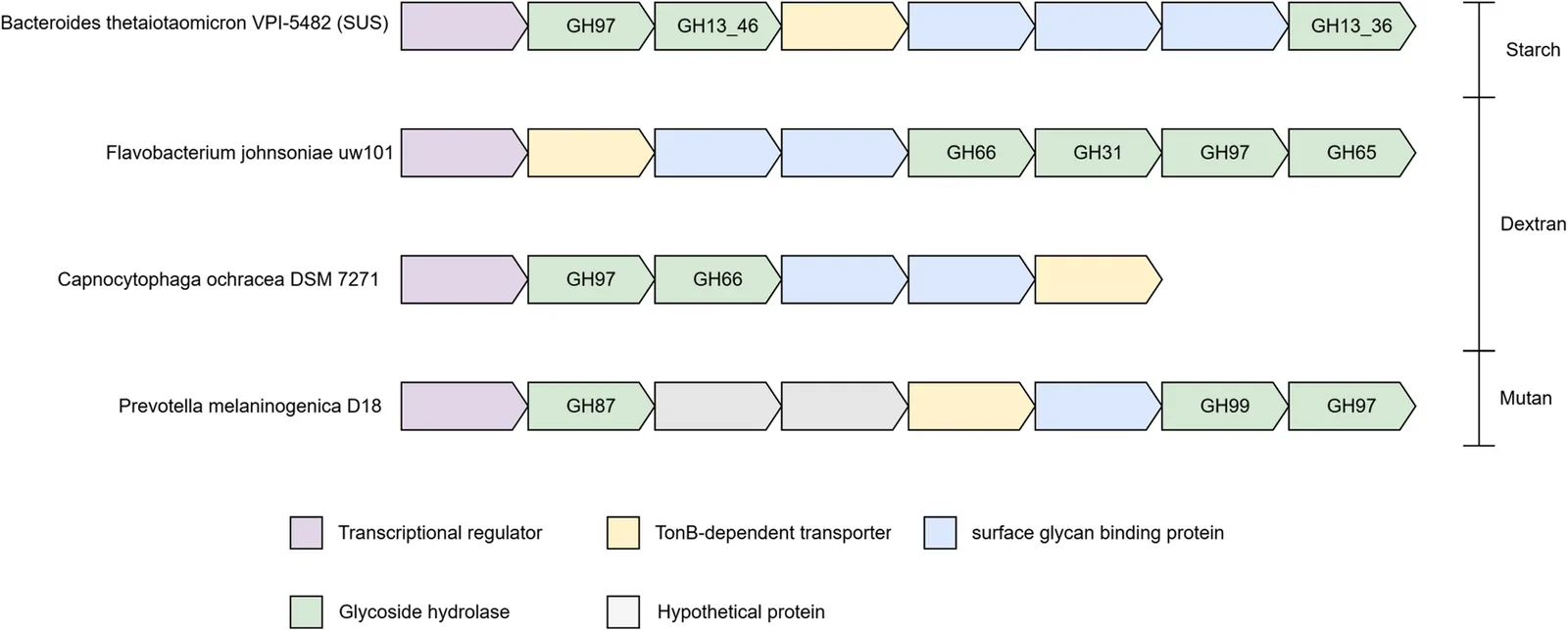
Organisation of various α-glucan polysaccharide utilisation loci; *B. thetaiotaomicron VPI-5482* starch utilisation locus, *F. johnsoniae UW101* Branched dextran utilisation Locus, *C. ochracea DSM 7271* potential branched dextran utilisation locus and potential *P. melaninogenica D18* branched mutan utilisation locus. Colours for genes are as follows: transcriptional regulator, purple; TonB-dependent transporter, yellow; surface sugar-binding protein, blue; glycoside hydrolase (labelled using CAZY nomenclature), green; hypothetical protein gray

The pPmPUL contained a SusR like transcriptional regulator, GH family 87 (PmGH87) enzyme, two proteins of unknown function—a short and a conserved hypothetical protein, respectively—a TonB-dependent transporter, single SusD like glycan binding protein, and enzymes placed into GH families 71/99 (henceforth PmGH99; see following section) and 97 (PmGH97) by automatic annotation. (Fig. 1). The PmGH87 and PmGH99 featured type II lipoprotein signal peptides and were localised to the extracellular membrane with high (0.9445) and low (0.4518) confidences, respectively. The two hypothetical proteins were localised to the outer membrane and periplasmic spaces—both with low confidence (0.5792) and (0.6407)—respectively. Whilst the GH97 featured a type 1 signal peptide and was confined to the periplasmic space with high confidence (0.9764).

### Predicted protein structures

**Fig. 2:**
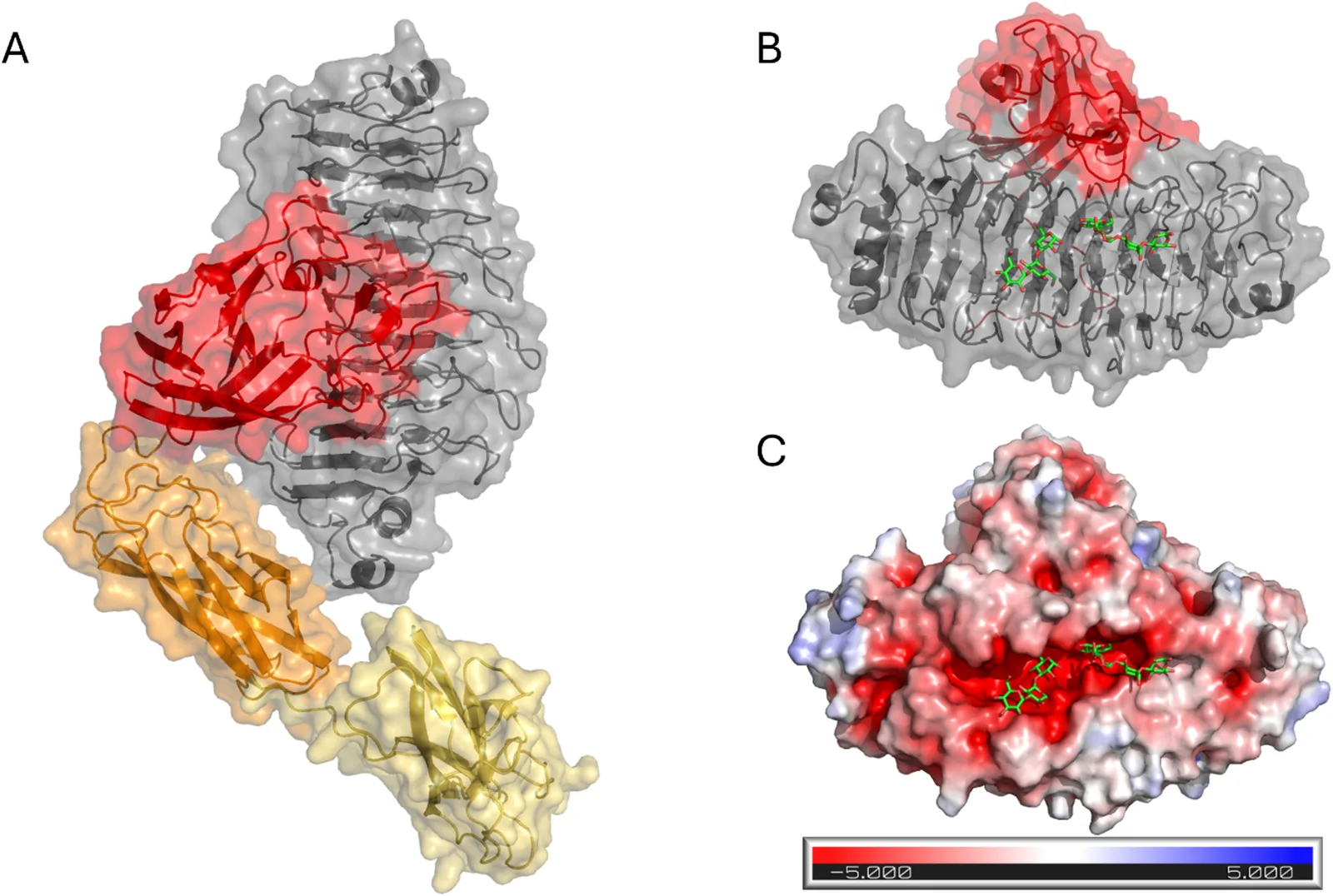
(**A**) Predicted model of PmGH87 3D structure demonstrating the N-terminal CBM domain (red), right-handed beta helix fold domain (grey), and two C-terminal β-sandwich domains (orange and yellow). (**B**) Rotated view of catalytic unit—comprising N-terminal CBM domain (Red) and right-handed beta helix fold domain (grey)—displaying the putative catalytic groove superimposed with α-(1→3)-glucooligosaccharides (lime) from the aligned crystal structure of *P. glycanilyticus* FH11 α-(1→3)-glucanase (Agl-FH1, PDB 6K0u). (**C**) electrostatic surface of PmGH87 where negatively charged regions are indicated in red and positively charged areas are colored blue; highlighting, the negatively charged catalytic cleft

Like other known GH87 members PmGH87 is a multimodular enzyme, sharing a conserved domain structure as follows: (Cortez et al. 2023; Itoh et al. 2020a, b; Yano et al. 2019) From N- to C-terminal, PmGH87 consists of a carbohydrate binding domain (CBM) which comprises a β-sandwich fold—and resembles a CBM35/6 or galactose-binding-like domain—a right-handed β-helix fold domain, and 2 β-sandwich, domains connected by long flexible linkers. (Fig. 2A) The CBM and β-helix domains are intimately associated with one-another, forming a single globular entity which functions as the catalytic unit with the CBM forming part of the wall of the catalytic-groove (Fig. 2B) and has been shown to be essential for catalytic activity (Itoh et al. 2020a, b; Yano et al. 2019; Shimotsuura et al. 2008). PmGH87 exhibits an extended, deep but solvent accessible and negatively charged groove which is assumed to be the catalytic cleft based on alignment with known crystallographic structures. The majority of catalytic groove is formed by extended loops of the β-helix domain.

**Fig. 3:**
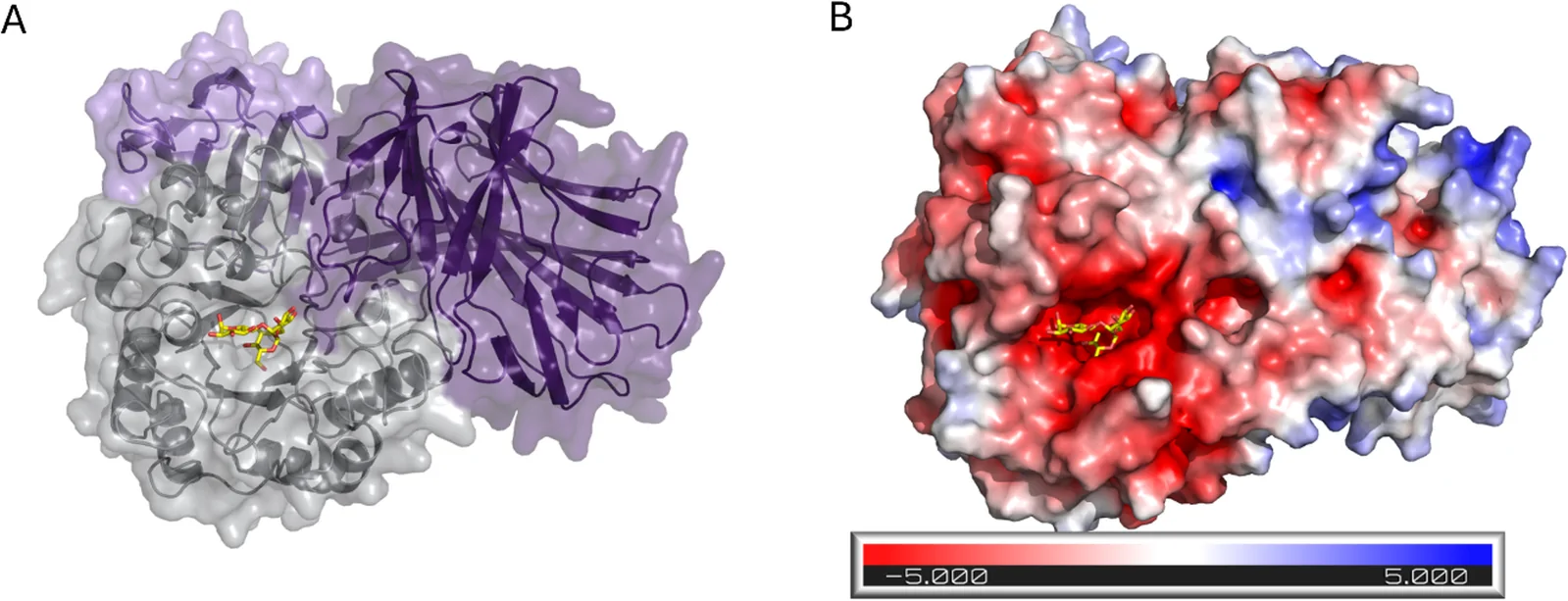
Predicted model of PmGH97 3D structure (**A**) showing 3 domain architecture: N-terminal domain β-sandwich domain (dark purple), catalytic domain TIM-barrel domain (grey), C-terminal β-sandwich domain (light purple). superimposed with isomaltotriose in the putative catalytic pocket from the aligned crystal structure of (FjGH97A, PDB 8WG2). (**B**) electrostatic surface of PmGH97 with negatively charged regions indicated in red and positively charged areas in blue, highlighting, the negatively charged putative catalytic pocket

PmGH97 exhibited an overall structure typical of GH97 enzymes consisting of three globular domains, an N-terminal beta-sandwich domain, central catalytic TIM-barrel domain, and C-terminal beta-sandwich domain (Nakamura et al. 2024, Kitamura et al. 2008), tightly associated with one another to form a compact structure. (Fig. 3) Of all GH97 members with known crystallographic structures PmGH97 shared the most identity with PspAG97A (41.96% Identity) (Li et al. 2016, He et al. 2016). Similarly to PspAG97A, it possesses a deletion of the loop corresponding to loop-N (G184-G204 in FjGH97) resulting in a wider and shallower active site pocket (Nakamura et al. 2024). In addition to the putative catalytic pocket the predicted structure of PmGH97 displayed an additional pocket of unknown function positioned adjacent to the catalytic active site (Figs. 3, S8).

**Fig. 4:**
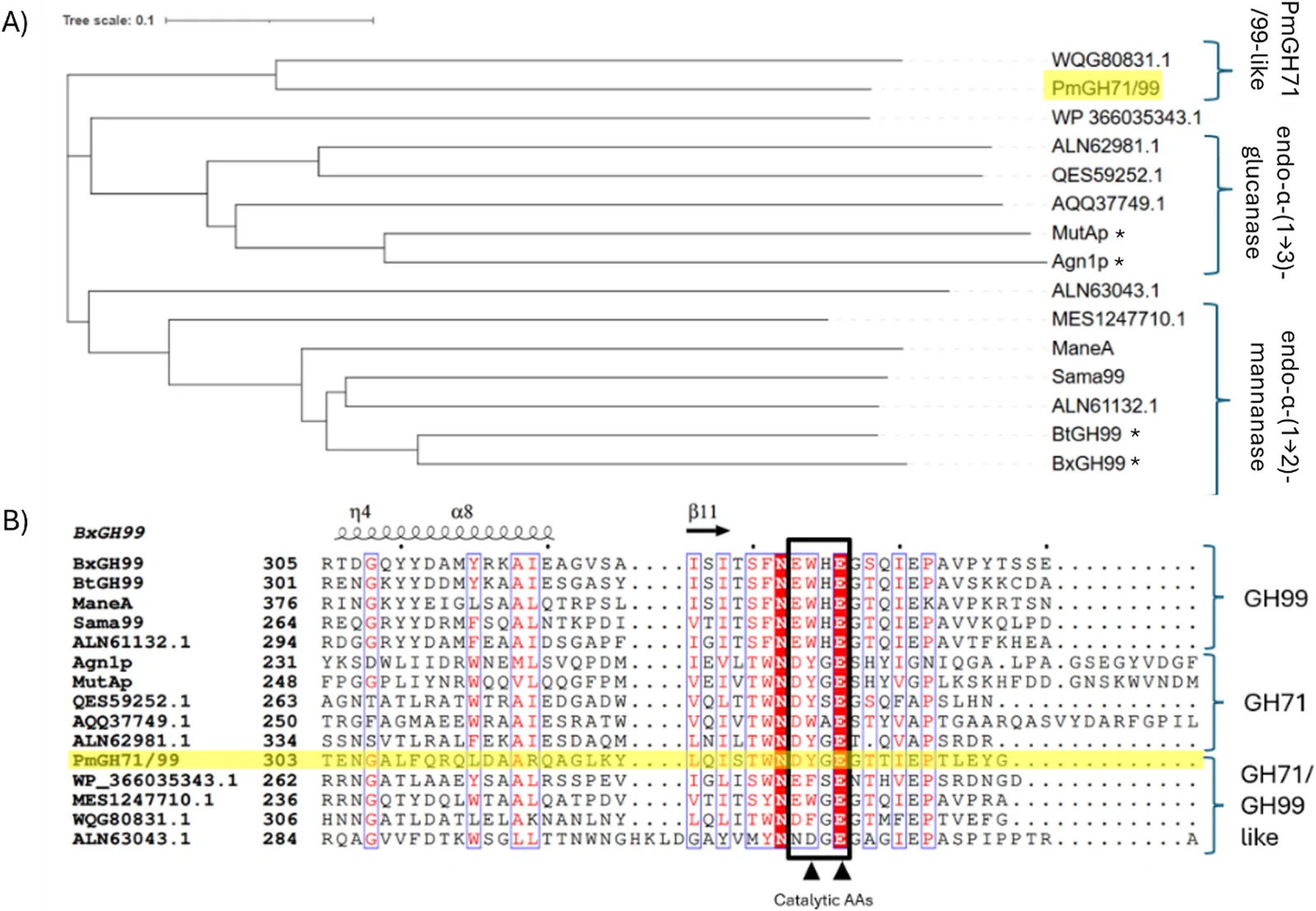
(**A**) Phylogenetic tree of enzymes showing sequence homology to PmGH99, grouped based on amino acid sequence similarity and labelled according to the activities of experimentally characterized representative members marked with (*), including endo-α-(1→3)-glucosidases (GH71) and endo-α-(1→2)-mannosidases (GH99). (**B**) Sequence alignment of enzymes with known activities from families GH71 and GH99, box shows conserved amino acid sequences, catalytic amino acids are marked with ▲, sequences were grouped based on automatic annotations in ncbi database

PmGH99 was assigned to the GH71/99 superfamily (Cd11575 GH99_GH71_like_3) by protein domain specific BLAST. Of proteins with known crystallographic structures PmGH99 had highest sequence similarity with *Bacteroides thetaiotaomicron* GH99 (BtGH99) 60% coverage with 22.96% identity score. However, it appears relatively distantly related to characterized members (Fig. 4A). Despite this, we decided to use the name PmGH99 for brevity and consistency with naming conventions, noting its higher sequence similarity with characterized GH99 members over GH71.

**Fig. 5:**
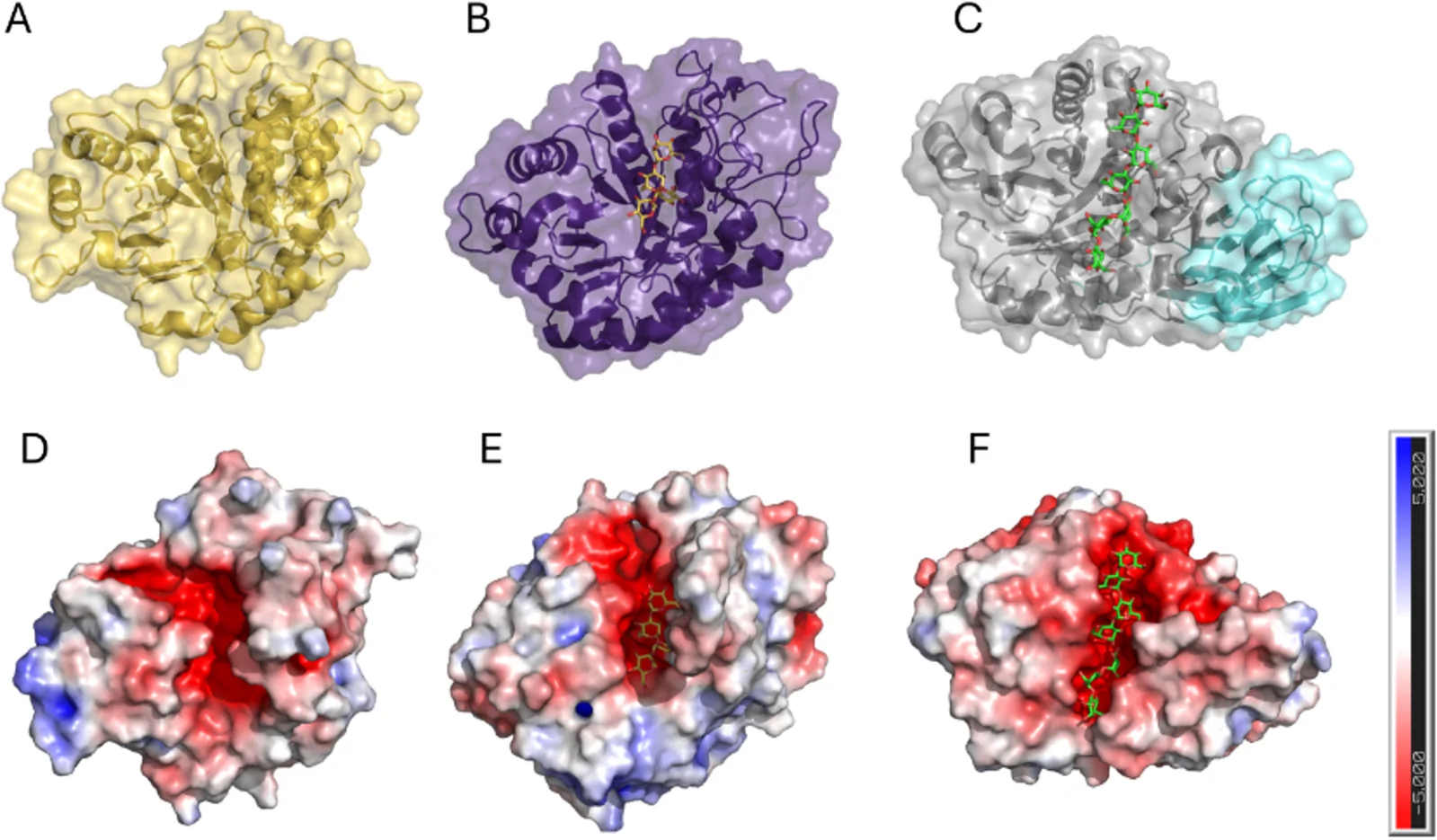
Comparison between aligned structures of (**A**) Predicted Structure of PmGH99 (yellow); (**B**) Crystal structure of BxGH99 (Purple) with α-mannan tetrasaccharide (yellow) bound to catalytic groove; (**C**) Crystal structure of the two domains of AnGH71C—Catalytic (grey) and C-terminal (cyan) domains—with α-(1→3)-glucooligosaccharides (lime) bound to the catalytic groove; (**D**, **E**, **F**) Represent the electrostatic surfaces of PmGH99, BxGH99 and AnGH71C, respectively, where negatively charged regions are indicated in red and positively charged areas are coloured blue. highlighting, in each case, the negatively charged catalytic cleft

PmGH99 consists of a single TIM barrel domain and exhibited a solvent accessible groove formed by extended loops—like other known GH99 structures. (Fig. 5A, B) Alternatively, fungal GH71 enzymes consist of an N-terminal TIM barrel domain and C-terminal beta sandwich domain (Fig. 5C) (Mazurkewich et al. 2025; Horaguchi et al. 2025). GH99 enzymes have the conserved catalytic sequence EWHE whilst GH71 members have the consensus sequence DYGE (Sobala 2023). PmGH99 has the DYGE consensus sequence (Fig. 4B).

### Enzymatic activity and substrate specificity assays

Initial substrate screening assays were performed against the polysaccharide substrates (soluble starch, *S. mutans* biofilm (WBF) and *Leuconostoc* dextran). Regarding the novel enzymes (PmGH97 and PmGH99), the observed activity was measurable but low even for extended incubation periods up to 24 h (Fig. S3A). However, PmGH97 displayed the highest activity against soluble starch with minor activity against dextran and little to no detectable activity against the biofilm substrate. As for PmGH99, the highest activity was observed against WBF. (Fig. S3A).

Strong activity of PmGH97 against the synthetic substrate pNP-α-Glc confirms α-glucosidase (EC: 3.2.1.20) activity. Therefore pNP-α-Glc was used in the subsequent assay to assess the effect of pH on enzymatic activity. The enzyme displayed an optimum pH of 6 with > 80 relative activity up to pH 7 (Fig. S5A). PmGH87 displayed high specificity for the lyophilized biofilm substrate (WBF) however its specific activity was also low (0.0903 ± 0.0073 U/mg)—although consistently higher than that of PmGH99 (0.0212 ± 0.0059 U/mg)—which obfuscated further kinetic profiling (Table S4). Nevertheless, the crude substrate was used in subsequent enzymatic assays to determine pH and temperature profiles, as previously reported (Cortez et al. 2023). The optimum pH and temperature for PmGH87 activity were 5.5 and 40 °C, respectively. The enzyme displayed a relatively broad temperature range with > 90% of activity between 35 and 45 °C and a sharp drop off above 50 °C. Likewise, WBF was used to determine the pH profile of PmGH99, which showed > 50% activity between pH 5–8 with a maximum at 7.16 (Fig. S5B). CoGH66, on the other hand, displayed robust activity against commercial dextran (60.35 ± 9.396 U/mg)—in agreement with the previous data (Cortez et al. 2023)—allowing for determination of kinetic parameters Vmax (57.667 ± 1.620 U/mg) and K_M_ (0.583 ± 0.045 mM) (Table S4). CoGH66 displayed the same optimum pH of 5.5 as PmGH87 and retained > 90% relative activity over a wide pH range (4.5–6.5), however, it displayed a more needle shaped temperature curve and a higher temperature optimum of 50 °C as reported by Cortez and collaborators (Cortez et al. 2023). 

To overcome the limitations encountered in kinetic analyses, particularly with the insoluble and impure substrate prepared from *S. mutans* biofilm, glucan fractions were extracted from biofilms to improve substrate definition and accessibility. Enzymatic activity was therefore reassessed against the extracts: water-insoluble (WIG) and water-soluble glucan (WSG), alongside the whole lyophilized biofilm (WBF), as described below.

**Fig. 6:**
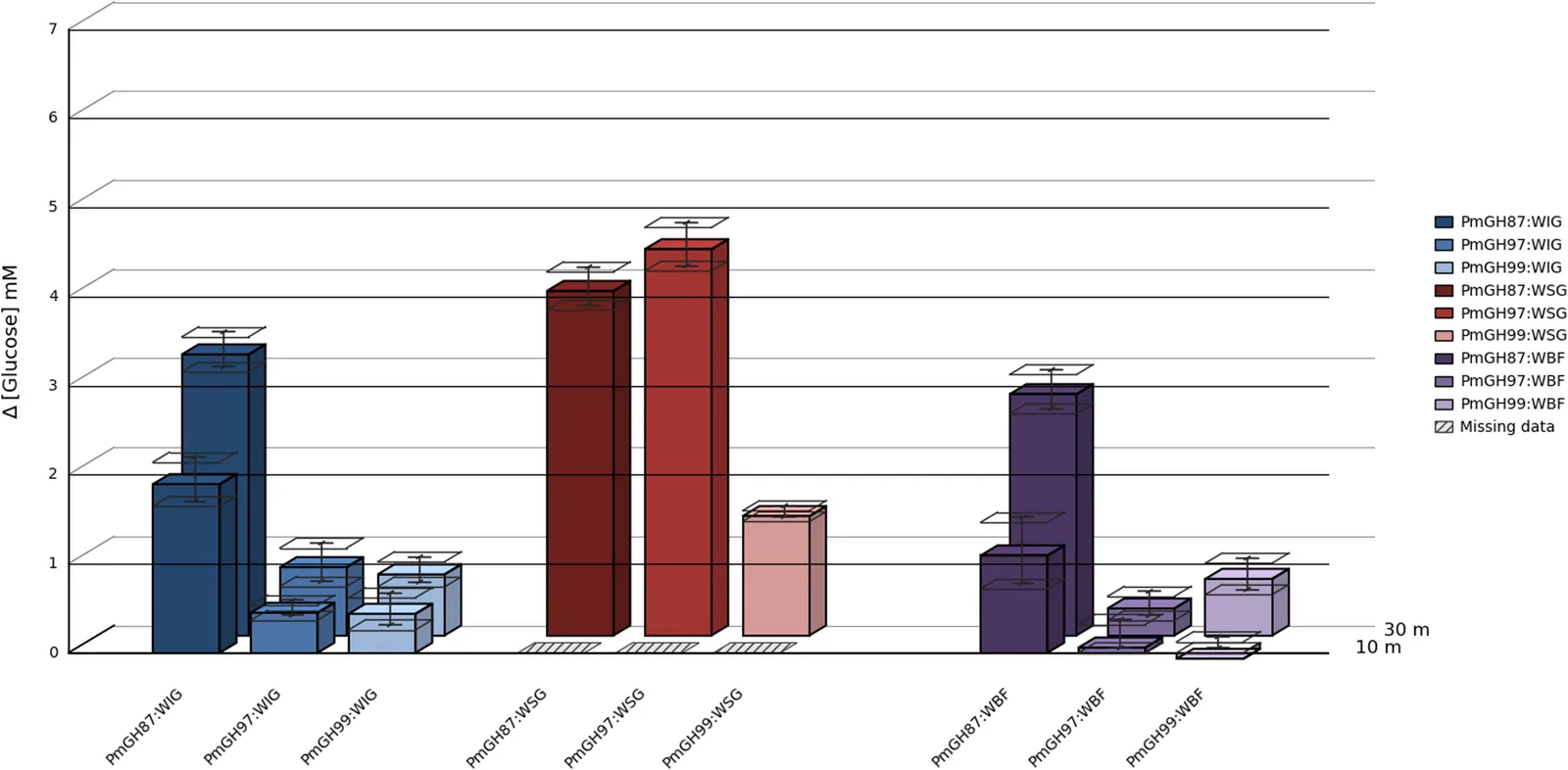
Time-dependent glucan-degrading activity of *P. melaninogenica* PUL enzymes against water-insoluble glucan (WIG), water-soluble glucan (WSG), and whole biofilm (WBF) fractions. Reducing sugar release (Δ glucose mM) was quantified by DNS method following incubation of substrates with PmGH87, PmGH97, and PmGH99. Reactions were performed with 1 mg/mL enzyme and 1% (w/v) substrate in MES buffer (50 mM, pH 6.0, 150 mM NaCl) at 40 °C with agitation (1000 rpm). Measurements were taken at 10- and 30-min. Bars represent mean values ± standard deviation

Overall, the reactions progressed as expected, displaying a clear time-dependent increase in product formation. All enzymes exhibited higher activity against the glucan extracts (WIG and WSG) compared to the whole lyophilized biofilm (WBF). Of enzymes applied to WIG, PmGH87 displayed the highest activity. In contrast, PmGH97 exhibited relatively low activity against WIG—comparable to PmGH99. All enzymes demonstrated enhanced activity against the WSG fraction. PmGH97 and PmGH87 displayed broadly similar activity profiles toward WSG (Fig. 6).

Given that PmGH99 shares highest sequence homology with characterized GH99 endo-α-(1→2)-mannanases, its potential role in the degradation of yeast-derived mannans was investigated. Activity was therefore assessed against whole *Saccharomyces cerevisiae* cells as a model substrate. No detectable activity was observed in the DNS assay for reaction mixtures containing PmGH99 and whole yeast cells at any incubation time tested, including extended incubations of up to 24 h, indicative of an absence of measurable reducing sugar release under the conditions employed.

### Effect of Enzymes on biofilm degradation

Next, we applied pPmPUL enzymes to in vitro grown *S. mutans* biofilms, both individually and in combinations, to determine their effects on biofilm degradation, and any potential synergism, evaluated via CV assays (see Materials and Methods: Biofilm degradation assays). Furthermore, we applied a positive control consisting of a previously explored and high performing heterologous combination of enzymes (PmGH87 and CoGH66) as described by Cortez and collaborators (Cortez et al. 2023) to further contextualize the results. Total enzymatic dose for combinations and the duration of application was fixed at 1 mg/ml and 4 h, respectively. Biofilms were preformed for 24 h prior to the experiments as previously described (see Materials and methods Biofilm degradation assays).

**Fig. 7:**
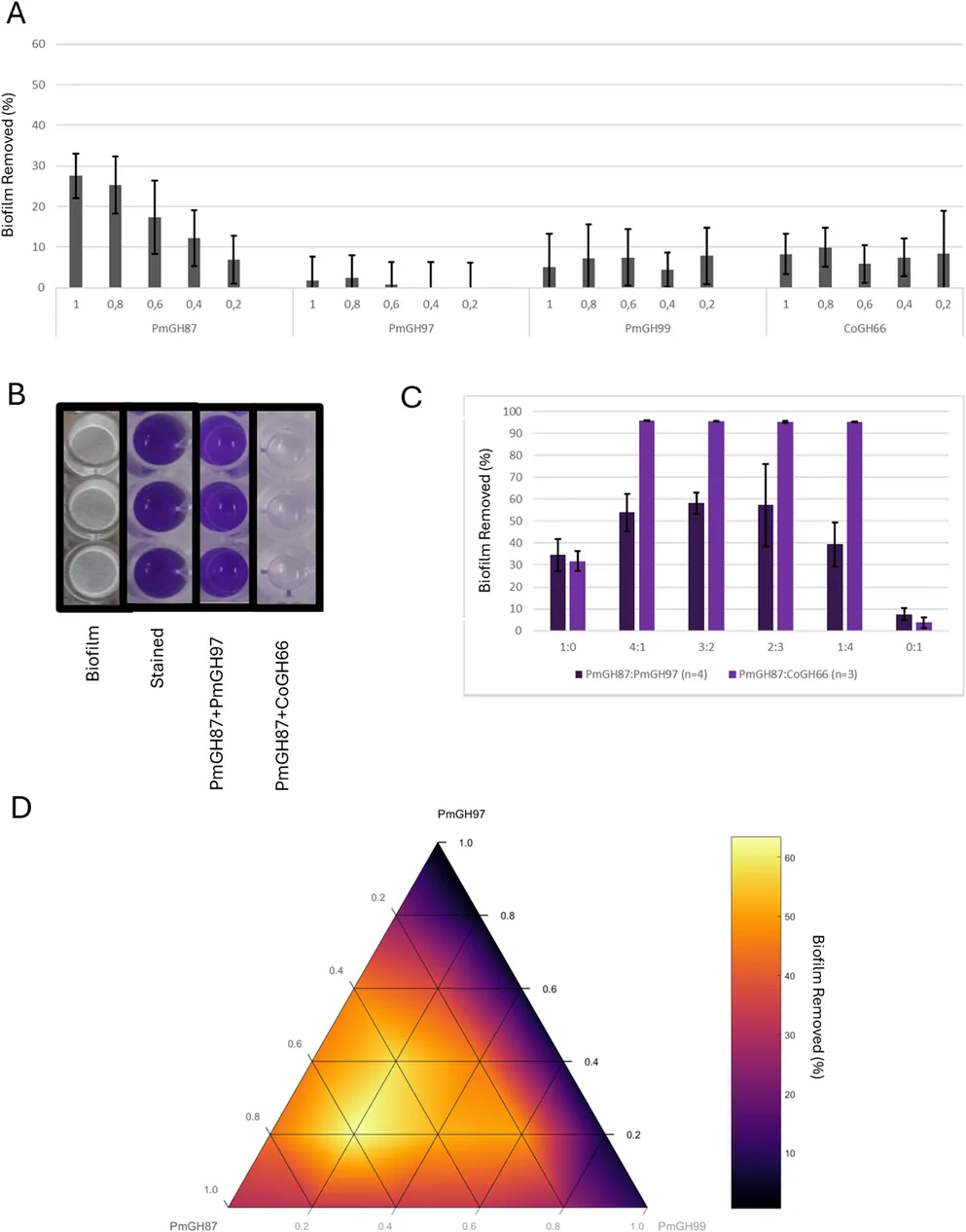
Results of Biofilm degradation experiments: (**A**,**C**) bar plots showing biofilm removal (%) with bars representing mean ± standard deviation of (**A**) enzymes applied individually at different concentrations with (*n* = 4), (**C**) binary combinations (with total enzymatic dose fixed at 1 mg/ml) of either PmGH87:PmGH97 (*n* = 4) or PmGH87:CoGH66 (*n* = 3), (**B**) Images of Biofilm in 96-well plate stained with crystal violet, (**D**) ternary contour plot of biofilm removal (%) of combinations of pPmPUL enzymes (with total enzymatic dose fixed at 1 mg/ml; *n* = 4). All experiments performed at 37 °C for 4 h in MES buffer (PH 6; final concentration: 50mM MES, 150mM NaCl). Experimental controls consisted of only MES buffer and the same volume of enzyme storage buffer (PH 7.5; final concentrations: 50 mM Tris, 200 mM NaCl) as the corresponding reaction. Experiments were performed on separate days, all biofilms were inoculated from cultures prepared from the same colony, standardized spectrophotometrically to an OD600 of 1.0 ± 0.05, diluted 1:100 in fresh biofilm media supplemented with sucrose (1% w/v) and grown for 24 h in 96-well plates prior to application of enzymatic treatments

The maximum biofilm removal effect of any individual enzyme (34.59 ± 3.81%) was achieved with PmGH87 (1 mg/ml), and the effect was dose dependent. (Fig. 7C) All other enzymes resulted in biofilm removal < 10% when applied alone (Fig. 7A). Ternary and binary combinations of pPmPUL enzymes containing PmGH87 and PmGH97 resulted in significantly enhanced biofilm removal (Fig. 7C–E) with a maximum observed value of (63.45 ± 29.73%) obtained with (3:1:1 PmGH87:PmGH97:PmGH99, total dose = 1 mg/ml). The inclusion of PmGH99 in combinations of pPmPUL enzymes appeared to result in a small increase in biofilm removal, however, large standard deviation does not allow to affirm statistical significance of this fact. (Figs. 7D, S9B)

Similar degradation values were observed with binary combinations of PmGH87 and PmGH97 with a maximum value of (58.18 ± 6.62%) obtained with (3:2 PmGH87:PmGH97, total dose = 1 mg/ml; Fig. 7C) as well as a synergy value of 3.36. In contrast, combinations of PmGH87 and CoGH66 achieved > 95% degradation within 4 h for all ratios tested (with synergy values between 2.84 and 5.65. (Fig. 7C)

### Confocal laser scanning fluorescence microscopy

**Fig. 8:**
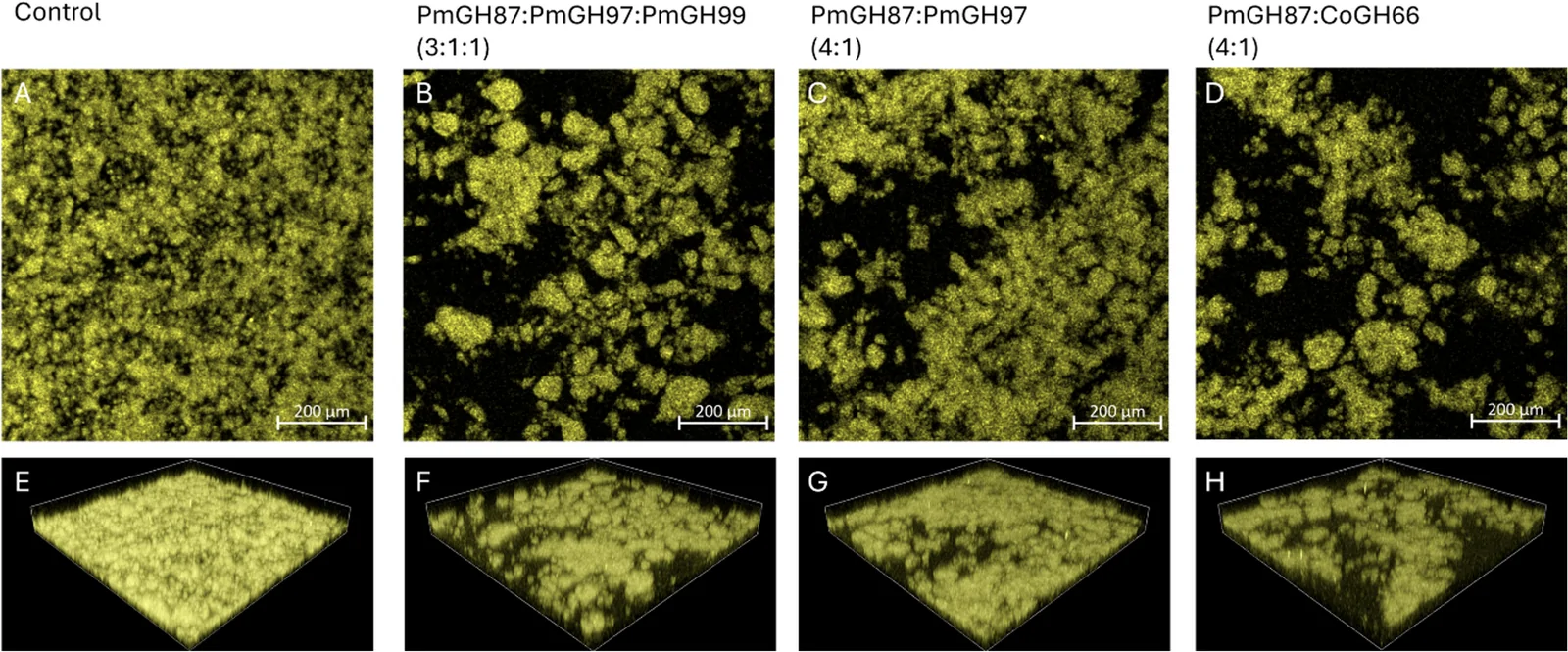
CLSM images of S. mutans Biofilm after experimental treatments: (**A**, **B**, **C**, **D**) 2D slices, (**E**, **F**, **G**, **H**) 3D reconstruction of biofilm. (**A**, **E**) control, (**B**, **F**) Best performing ternary combination (3:1:1 PmGH87:PmGH97:PmGH99), (**C**, **G**) Binary mixture (4:1 PmGH87:PmGH97), (**D**, **H**) Positive control (4:1 PmGH87:CoGH66). All experimental treatments utilized a fixed total dose of 1 (mg/ml). All experiments performed at 37 °C for 2 h in MES buffer (PH 6; final concentration: 50mM MES, 150mM NaCl). Experimental controls consisted of only MES buffer and the same volume of enzyme storage buffer (PH 7.5; final concentrations: 50 mM Tris, 200 mM NaCl) as the corresponding reaction. Experimental treatments were performed in parallel to biofilms formed in the same 24-well plate, all biofilms were inoculated from cultures prepared from the same colony, standardized spectrophotometrically to an OD600 of 1.0 ± 0.05, diluted 1:100 in fresh biofilm media supplemented with sucrose (1% w/v) and grown for 24 h prior to application of enzymatic treatments

To visualize changes in biofilm architecture and to confirm results obtained with CV assays CLSM images were taken using the endogenous fluorescence of the biofilms. Three different high performing experimental treatments were chosen (each consisting of a mixture of enzymes with total dose fixed at 1 mg/ml) with the aim of elucidating on differences in biofilm removal effect observed during CV assays. Experimental treatments consisted of the best performing ternary mixture of pPmPUL enzymes (3:1:1 PmGH87:PmGH97:PmGH99), and two binary mixtures—either (4:1 PmGH87:PmGH97) or the positive control (4:1 PmGH87:CoGH66)—were applied to wells with pre-grown *S. mutans* biofilm in a 24-well microtiter plate for 2 h at 37 °C. The highest visible degradation was achieved with the mixture (4:1 PmGH87:CoGH66). This effect was pronounced, removing almost all biofilm adhered to the bottom of the well within 2 h of treatment (Fig. S4D, H, L). Representative slices of the remaining biofilm also show a greater degree of fragmentation of the biofilm matrix in comparison to other experimental treatments (Fig. 8D, H). Of experimental treatments consisting of pPmPUL enzymes the binary (4:1 PmGH87:PmGH97) and ternary (3:1:1 PmGH87:PmGH97:PmGH99) mixtures tested performed similarly. A higher degree of fragmentation was observed with the ternary combination containing PmGH99, (Fig. 8B, F) however the binary combination without PmGH99 appeared to result in a more homogenous reduction in biofilm thickness (Fig. 8G).

## Discussion

Although we did not experimentally characterise the pPmPUL, the existence of a *P. melaninogenica* PUL specified for the catabolism of MS biofilms is plausible. *Prevotella* species are frequently encountered in oral biofilms dominated by *Streptococcus* (Rikvold et al. 2023). Furthermore, the ability to modify or degrade biofilm EPS produced by MS may confer a significant evolutionary advantage, potentially aiding during initial colonisation and eventual dispersion and escape from the biofilm. The EPS also represents a significant potential metabolic resource.

The pPmPUL displayed novel structure in terms of its GH composition. To date, no known PUL containing (GH87, GH97 and GH99) has been described or characterized in the literature.

GH97 members are frequently encountered in PUL, however, they are not always present (Nakamura et al. 2023). Known members are frequently localized to the periplasmic space where they act against the digestion products of other PUL enzymes to release simple sugars for use by the bacteria. GH99 members have been described within PUL architectures specialized for the utilization of yeast mannan (Cuskin et al. 2015). The phylogenetic placement and activity of PmGH99 in context of characterised GHs is discussed in more detail in a later section. More generally, to the best of our knowledge, no PUL containing GH87 has been experimentally determined or characterised.

All experimentally characterised GH87 members encompass either mutanase (endo-(1→3)-α-glucanase; EC 3.2.1.59) or mycodextranase ((1→3)-(1→4)-α-D-glucan 4-glucanohydrolase; EC 3.2.1.61) activity (Itoh et al. 2020a, b; Yano et al. 2019; Shimotsuura et al. 2008; Okazi et al. 2001). Notably, mycodextranases display no activity against linear α-(1→3)-linked chains. Therefore, PmGH87 activity against *S. mutans* biofilm-derived substrates, which contain both α-(1→3)- and α-(1→6)-linkages—but not against dextran (α-(1→6)-linked)—confirms α-(1→3)-hydrolysis and hence mutanase activity. PmGH87 displayed relatively low specific activity (0.0903 ± 0.0073 U/mg) against a crude substrate prepared from lyophilized *S. mutans* biofilm, however this can be attributed at least in part to the impure nature of the substrate used in the assays. Indeed, the enzyme did display enhanced activity against the partially purified extracts (WSG and WIG) prepared from the crude biofilm substrate. However, the overall activity remained low. Comparable specific activity values (0.075–0.097 U/mg) have been reported for other mutanase enzymes (Villalobos-Duno et al. 2013). Furthermore, several reports note reduced substrate affinity and activity specifically against insoluble α-(1→3)-glucans (Shimotsuura et al. 2008; Pleszczyńska et al. 2012; Horaguchi et al. 2023a, b). To overcome these effects, several groups utilise chemical or physical methods to artificially solubilize insoluble substrates prior to enzymatic assays (Shimotsuura et al. 2008; Grün et al. 2006; Villalobos-Duno et al. 2013; Horaguchi et al. 2023a, b). 

Of all GH97 members with known crystallographic structures PmGH97 shared the most identity with PspAG97A (41.96% Identity) an exo-dextranase with wide substrate specificity isolated from the marine bacterium *Pseudoalteromonas sp. K8* (Li et al. 2016, He et al. 2016). PspAG97A was able to hydrolyse various linkage types in glucooligosaccharides of diverse sizes, including α-(1→4)- and α-(1→6)-linkages, with a preference for large chain α-(1→6)-linked glucans, i.e. dextrans. Furthermore, it efficiently hydrolysed the synthetic substrate pNP-α-Glc (Li et al. 2016). 

PmGH97 activity against commercial dextran demonstrates that although it is capable of hydrolysing α-(1→6)-linkages in extended chains, this is not its preferred substrate. The enzyme displayed higher activity against soluble starch over a 24 h period (Fig. S3)—CoGH66 on the other hand, readily hydrolyses commercial dextran with high efficiency. Furthermore, strong activity of PmGH97 against pNP-α-Glc confirmed α-glucosidase activity. As exolytic enzymes, α-glucosidases cleave sugars from the terminal non-reducing ends of oligosaccharide substrates, recognizing α-(1→4)- and α-(1→6)-linkages, often with reduced efficiency for the latter. Characterised members usually display preference for short chain oligosaccharides over polysaccharide substrates (Nakamura et al. 2024). 

Although we did not test the ability of PmGH97 to hydrolyse α-(1→3)-linkages in defined substrates, its activities against WBF and WIG were low. The enzyme displayed a preference for WSG over WIG and WBF likely due, at least in part, to the soluble nature of the substrate and potentially to a higher proportion of α-(1→6)-linkages. Some GH97 members are able to hydrolyse α-(1→3)-linkages in oligosaccharide substrates (Nakamura et al. 2023). 

The GH71 family is most closely related to GH99 in terms of sequence similarity (Huo et al. 2017; Sobala 2023) GH family 71 was initially discovered in fungi and originally believed to be unique to them (Pleszczyńska et al. 2017). however, their presence in bacteria was later confirmed and they are now known to be common in members of the *Actinobacteria* taxon (Costa et al. 2020). Known GH99 enzymes consist of a single TIM barrel domain whilst fungal GH71 feature an additional C-terminal β-sandwich domain in conjunction with the TIM barrel (Mazurkewich et al. 2025; Horaguchi et al. 2025). To the best of our knowledge, no bacterial GH71 enzymes have been characterised biochemically or structurally at the time of writing.

Known GH99 activities include endo-α-(1→2)-mannosidases (EC 3.2.1.130) and endo-α-(1→2)-mannanases (EC 3.2.1.198) (Thompson et al. 2012; Sobala et al. 2020). In contrast, all biochemically characterized GH71 enzymes belong to the mutanase (EC 3.2.1.59) activity class (Mazurkewich et al. 2025; Horaguchi et al. 2025). α-Mannan degrading enzymes may be useful in the efficient production of pro-biotic compounds and research materials for novel applications.

PmGH99 was assigned to the GH71/99 superfamily (Cd11575 GH99_GH71_like_3) by protein domain specific blast. Of proteins with known crystallographic structures PmGH99 had highest sequence similarity with *Bacteroides thetaiotaomicron* GH99 (BtGH99) 60% coverage with 22.96% identity score. However, it appears relatively distantly related to characterized members, possibly diverging from a common ancestor before the GH71/99 split (Fig, 4 A). PmGH99 consists of a single TIM barrel domain—like other known GH99 structures—however, it features the DYGE catalytic motif, characteristic of GH71 enzymes (Sobala 2023). 

Considering PmGH99 shared the highest sequence and structural similarity with GH99 endo-α-(1→2)-mannanases, we hypothesized that it may play a role in mannan degradation in mixed kingdom streptococcal-fungal biofilms. To test this, we performed assays against commercial whole *S. cerevisiae* cells as described in (Materials and Methods: Enzymatic assays). Notably, no activity was detected under the assay conditions employed, even for extended incubation times of up to 24 h. These findings provide evidence counter to the hypothesis that the pPmPUL is specifically adapted for degradation of yeast-derived components in mixed streptococcal–yeast biofilms. If PmGH99 belonged to such a PUL and was responsible for degradation of the yeast derived matrix components, activity against whole *S. cerevisiae* cells would be expected, considering that the major component of *C. albicans* biofilms—comprising α-(1→2)-decorated, α-(1→6)-linked mannopyranose chains—is also present in considerable amounts in *S. cerevisiae* cell walls (Pierce et al. 2017; Yousefi 2023). 

Considering the low specific activities observed for PmGH99 against all substrates tested it is likely that its preferred substrate remains yet undetermined. However, the enzyme showed low but measurable specific activity (0.0212 ± 0.0059 U/mg) against the crude biofilm substrate. This is notable because no such activity has been described previously for GH99 members. A potential concern when utilizing polysaccharides derived from bacterial culture culture—relevant to the *S. mutans* derived substrates—is contamination with components of the growth media, such as yeast mannans present in yeast extract (Rangarajan et al. 2013). However, it is worth noting that α-mannans are mainly present in the cell walls of *S. cerevisiae* and should only be present in minor quantities in soluble yeast extract. Furthermore, considering the concentration of yeast extract used in the media (1.5% w/v), that α-mannan represents approximately (7–10% w/w) of total yeast dry mass (Yousefi 2023), and the maximum concentration of yeast cells used in the assay (10% w/v), If the observed activity were indeed due to contamination with yeast mannan, then we would likely also observe activity in the assay with whole yeast cells, which was not observed. It is perhaps worth noting that other polysaccharides besides glucans may be extracted from *S. mutans* cells within the biofilm under the conditions applied. Both rhamnoglucan and glycogen-like polysaccharides, have been observed in extracts prepared by sonication or alkali treatment (dos Santos Ré et al. 2024). 

The higher activities observed against the glucan extracts (WIG and WSG) compared to the whole lyophilized biofilm (WBF), likely reflect higher relative concentrations or accessibility of the substrates. In particular, all enzymes demonstrated higher activity toward WSG which may pertain to increased accessibility of the soluble substrates. In assays against WBF, PmGH87 exhibited higher activity than PmGH99, while PmGH97 showed little detectable activity. Similarly, PmGH87 displayed the highest activity against WIG of all enzymes tested. This observation was expected and is consistent with its proposed role in the cleavage of main-chain α-(1→3)- glycosidic bonds in the insoluble biofilm matrix. Surprisingly, PmGH97 displayed comparable activity to PmGH99 toward WIG.

As stated previously, oral biofilms produced in situ are significantly composed of glucans which are produced extracellularly by gtf enzymes largely derived from streptococcus species (Klein et al. 2015; Bowen and Koo 2011). For example, *S. mutans* produces several distinct gtfs, each producing distinct glucan products, differing in linkage structure and chemical and physical properties.

MS glucans can be broadly divided into water soluble (WSG) and water insoluble (WIG) fractions, however it is important to note that each of these categories may represent a range of molecules rather than distinct chemical entities. WIGs form the major structural components of MS biofilms while WSGs serve mainly to modify its properties and possible as a reserve source of carbohydrate. It has been proposed that the WIG present in oral biofilms consist mainly of α-(1→3)-linkages in the backbone, with a small amount of α-(1→6)-linkages, most likely comprising branches (Bowen and Koo 2011; Cortez et al. 2023). However, heterologously expressed gtfs from *S. mutans (GS-5)* were observed to produce several distinct WIGs *in vitro.* These WIGs contained α-(1→3)- and α-(1→6)-linkages in comparable proportions. (48% to 55% α-(1→3)-linked units) with both linkage types present in the backbones as well as the branches of representative WIGs (Ernst et al. 2024). 

Interestingly, some oral *Streptococci* such as *Streptococcus salivarius* have been observed to produce WIG consisting entirely of linear α-(1→6)-linkages (Ernst et al. 2024). The precise glucan composition of biofilms produced by MS specimens may vary across strains and differing experimental conditions. In fact, an almost continuous distribution of glucans with varying linkage proportions has been observed for different strains of *S. mutans*, alone (Trautner et al. 2009). 

Besides glucans, the EPS of oral biofilms comprise proteins, eDNA and fructans (Bowen et al. 2018; Klein et al. 2015; Bowen and Koo 2011). Fructans represent only 1–2% of in-situ oral biofilms and are produced extracellularly via the action of fructosyltransferase (ftf) enzymes on sucrose—although, like gtfs, they have been shown to accept other oligosaccharides substrates, allowing for the synthesis of fructans and the assembly of a biofilm matrix under relatively low sucrose conditions (Nagasawa et al. 2017)—they represent a reserve source of carbohydrate, potentially prolonging periods of heightened metabolism and acid production following sucrose exposure (Bowen et al. 2018). Despite representing a minor proportion of the biofilm EPS, they may be structurally significant particularly under low sucrose conditions, where their interaction with eDNA promotes the assembly of the biofilm matrix (Nagasawa et al. 2017). Furthermore, they may alter the physical properties of the biofilm matrix, similar to WSG. Therefore, they may represent interesting additional targets for the enzymatic degradation of oral biofilms.

The presence of both α-(1→3)-, α-(1→6)-linkages in *S. mutans* derived WIG explains why combinations of mutanase (PmGH87) and either endo-dextranase (CoGH66) or α-glucosidase (PmGH97) resulted in superior biofilm degradation than any of the enzymes alone. Both endo-dextranase and α-glucosidase have been shown capable of hydrolysing α-(1→6)-linkages, while mutanase is specific to α-(1→3)-linked glucans. Combinations of PmGH87 and CoGH66 outperformed PmGH87 and PmGH97, achieving > 95% degradation within 4 h, as opposed to (58.18 ± 6.62%), and resulting in the highest visible degradation as observed via CLSM, removing almost all biofilm adhered to the bottom of the wells within 2 h of treatment.

In general, studies employing mutanases and endo-dextranases against in vitro-grown oral biofilms demonstrate that their combination acts synergistically to enhance degradation efficiency, with biofilm removal reaching between 70 and 95% within 4 h (Del Rey et al. 2024; Pozelli Macedo et al. 2024; Pleszczyńska et al. 2017; Cortez et al. 2023; Macedo et al. 2026). The increase in nigerose (α-(1→3)-linked, gluco-disaccharide) production observed in combinations of the two enzymes demonstrates that the cleavage of α-(1→6)-linkages by endo-dextranase facilitates the access of mutanase to its substrate resulting in synergistically enhanced biofilm degradation (Cortez et al. 2023). Specifically, studies employing combinations of PmGH87 and GH66 endo-dextranases achieve 80–95% removal within 4 h (Cortez et al. 2023 ; Pozelli Macedo et al. 2024; Macedo et al. 2026). Endo-dextranase activities can be found across various MOs and GH families and may differ in their ability to tolerate cross-linkages and other chain modifications (Pozelli Macedo et al. 2024; Sugiura et al. 1973; Drula et al. 2022). Differences in biofilm removal effect observed may be partly due to differences in the activity, stability under assay conditions and substrate preferences of individual endo-dextranases, which warrants further study. Other combinations of enzymes have been tested against in vitro and in situ grown oral biofilms with varying degrees of success (Nielsen et al. 2024; Macedo et al. 2026). 

In contrast to the significantly enhanced biofilm removal observed with combinations of PmGH87 and GH66 endo-dextranases, no such synergy was observed with combinations of PmGH87 and an exo-dextranase from GH family 13 (DexB) (Macedo et al. 2026). Biofilm removal remained below 20%, only exceeding 50% when combined with a carbohydrate esterase (EcPgaB) from the CE4 family. Interestingly, no additional synergistic effect was observed for combinations of PmGH87, endo-dextranase (DexA), and EcPgaB (Macedo et al. 2026). The authors note that although esterases have been reported to enhance the eradication of several bacterial biofilms, their specific role in this effect remains to be elucidated. The effect was attributed speculatively to the degradation of the bacterial structures themselves, within the biofilm—via the targeting of peptidoglycan present in the cell walls—which may enhance access of exo-dextranase to the terminal sugars of glucans, facilitating hydrolysis (Macedo et al. 2026). Substrate preferences aside, α-glucosidases (EC: 3.2.1.20) which successfully cleave the terminal α-(1→6)-linkages in extended glucan chains perform a reaction equivalent to exo-dextranase (EC: 3.2.1.70). It is worth noting that although we observed greater biofilm removal with combinations of PmGH87 and PmGH97, than reported for PmGH87 and exo-dextranase, it did not exceed (58.18 ± 6.62%).

Interestingly, in vitro studies involving both mixed species biofilms grown in vitro and in situ grown biofilms show comparable degradation effect (~ 70%) by combinations of mutanase and either endo-dextranase or β-glucanase (Nielsen et al. 2024). Both endo-dextranase and β-glucanase were incapable of significant biofilm removal alone (< 15%) and showed no synergy when applied together. Of all enzymes tested individually, mutanase was the only to display substantial biofilm removal (> 40%) in line with our results. Furthermore, regarding in vitro biofilms, a broad selection of binary combinations including mutanase—and either (endo-dextranase, β-glucanase, cellulase, amylase or DNAse I) but not lipase—were observed to display improved biofilm removal compared to mutanase alone. Tertiary and higher combinations of enzymes did not display any statistically significant improvement in comparison to the best binary combinations. The referenced in vitro biofilms were inoculated from saliva samples collected from healthy volunteers and were not characterized in terms of species make-up or EPS composition. In contrast to results obtained from in vitro biofilms, mutanase was not necessary for substantial degradation of in situ biofilms. On the other hand, the inclusion of DNAse and lipase was with great effect and the best performing combination contained (endo-dextranase, β-glucanase, cellulase, DNase and lipase) and reduced the biofilm volume by 71% (*p* = 0.0062). Notably, this formulation did not contain mutanase and had only a moderate effect on *in vitro grown* biofilms (28% reduction), highlighting potential differences between biofilms formed in vitro vs. in situ. Conversely, in another study, the inclusion of DNase I (pancreatic bovine deoxyribonuclease A; Sigma-Aldrich) with PmGH87 mutanase and an endo-dextranase did not result in enhanced removal of *S. mutans* biofilm (Pozelli Macedo et al. 2024). However, eDNA in oral biofilms may adopt non canonical structures, which are resistant to degradation by DNase I, necessitating the use of alternative enzymes (e.g. DNase A) or chemical adjuvants to achieve substantial degradation (Evans et al. 2025). 

In our assays, despite demonstrating activity against substrates prepared from *S. mutans* biofilm, PmGH99 was insufficient in exerting substantial biofilm degradation alone, performing about as well as CoGH66 (Fig. 7A). It did not seem to enhance the biofilm removal effect of PmGH87 (Figs. 7D, S7A). Its inclusion with PmGH87 and PmGH97 in ternary combinations of pPmPUL enzymes may result in a small additional positive effect. However, noting a large standard deviation it is currently difficult to separate this relationship from experimental noise (Figs. 7D, S9B). Furthermore, its function in biofilm degradation is not clear, motivating further study.

The effectiveness of antimicrobial treatments is limited by their ability to penetrate into the biofilm matrix. Consequently, various in vitro studies have observed antimicrobial activity mainly at the surface of *S. mutans* biofilms (Ren et al. 2019; Cardoso et al. 2011; von Ohle et al. 2010; Koo et al. 2017). Emerging evidence demonstrates that treatments targeting the removal of the biofilm matrix—including enzyme-based approaches—may enhance the effectiveness of antimicrobial treatments against disease-associated oral bacteria (Ren et al. 2019; Rogers 2014). Comparable to results obtained with combined enzymatic biofilm degradation and antibiotic treatment against other clinically relevant MOs (Silva et al. 2025; Dabul et al. 2025; Cruz 2026). Beyond antimicrobials, biofilm degradation alone may be sufficient to enhance the clearance of harmful MOs from the oral cavity, and to directly attenuate cariogenesis by removing acid and fermentable substrate in contact with the enamel.

Several limitations of the present study should be acknowledged. First, the time required for breaking down the disease-associated biofilm is the major limitation of enzyme-based technologies. In addition, the duration required for significant biofilm removal should be sufficiently short to achieve efficacy within the period over which the enzymes remain active under the intended application conditions and treatment modality (such as oral cavity conditions, for example). Furthermore, oral biofilms are complex multispecies communities comprising hundreds of interacting microorganisms, which vary between individuals and undergo temporal shifts in response to changing environmental conditions. Consequently, the susceptibility of clinically relevant biofilms to enzymatic degradation may differ from that observed in the present study.

## Conclusions

Here we present, a predicted PUL from the microorganism *P. melaninogenica* (pPmPUL), which demonstrated novel structure in terms of GH composition. The pPmPUL contained a GH each from families 87, 97 and attributed to the 71/99 superfamily. Combinations of the pPmPUL enzymes were effective at removing S. mutans biofilm and performed better than enzymes applied individually. However, combinations of pPmPUL enzymes were less effective than of PmGH87 and CoGH66 which demonstrated > 95% biofilm removal within 2–4 h depending on assay conditions. Biofilm degradation effect was further confirmed via CLSM imaging. The results presented here may aid in the development of new enzyme-based therapeutics for the treatment of oral disease, by targeting the removal of cariogenic biofilms. Additionally, the present study also demonstrates a novel strategy for identifying combinations of enzymes that may display synergism and enhanced degradation efficiency against clinically relevant biofilms, more generally.

Results of biofilm degradation assays with pPmPUL enzymes demonstrate that the inclusion of PmGH99 in the formulation results in a small positive effect—this is in line with the observed activity against *S. mutans* derived substrates—however, noting a large standard deviation it is currently difficult to affirm statistical significance, motivating further testing. Furthermore, the preferred substrate of PmGH99 remains to be determined. Preliminary results point towards α-glucan hydrolase activity, which is novel for members of the GH99 family. More generally, further study is required to address the paucity of information on the subset of GHs automatically annotated as 71/99-like and on bacterial GH71 enzymes.

Differences in biofilm degradation observed with combinations of PmGH87 mutanase and various endo-dextranases may reflect differences in the properties of the endo-dextranases and hence, may highlight the superiority of specific enzymes relative to others, in this context. Likewise, studies performed under standardized conditions comparing the effect of various mutanases—particularly across different families such as GH71—may be useful in affirming those with greater biofilm removal efficiency or other desirable properties suitable to their application in the treatment of oral disease, including stability under application conditions—such as those found in the oral cavity—and during long-term storage, ease of expression, ability to bind to the biofilm and a preference for liberating larger oligosaccharides over simple sugars from the substrate, the latter being more readily fermentable and hence acidogenic.

In vitro testing is useful because of its speed and reproducibility, facilitating the identification of novel targets. However, considering the species diversity of the human oral microbiome, its variability between individuals, and both the importance and ubiquitousness of polymicrobial interactions, future studies may benefit from the use of either mixed species oral biofilms or biofilms grown in situ. Furthermore, there is substantial evidence in vitro for the effectiveness of combinations of mutanase and endo-dextranase in degrading oral biofilms, however, further clinical investigation is required to evaluate their potential for application in oral care.

## Supplementary Information

Below is the link to the electronic supplementary material.

Supplementary Material 1

## Data Availability

No datasets were generated or analysed during the current study.
