# Supplementary material for: Enzymatic tools for the treatment of caries-associated biofilms: Investigating the enzymes of a putative polysaccharide utilization locus from Prevotella melaninogenica and their application against Streptococcus mutans biofilm
Source: World J Microbiol Biotechnol. 2026 Jul 29;42(8):435. doi: 10.1007/s11274-026-05104-8 (PMC13421300; doi:10.1007/s11274-026-05104-8)
Supplement: Supplementary file 1 — Supplementary Material 1 [file 11274_2026_5104_MOESM1_ESM.pdf]

Supplementary Information

Enzymatic tools for the treatment of caries-associated biofilms: Investigating the enzymes of a putative polysaccharide utilization locus from *Prevotella melaninogenica* and their application against *Streptococcus mutans* biofilm

Dylan James Erasmus[1], Adelita Carolina Santiago[1], Sebastião Pratavieira[1], Neil Thomas Stacey[2] and Igor Polikarpov[1,#]

[1]São Carlos Institute of Physics, University of São Paulo, Avenida Trabalhador São-carlense, 400, Parque Arnold Schimidt, 13566-590 São Carlos, SP, Brazil

[2]University of the Witwatersrand, Johannesburg (Wits), 1 Jan Smuts Avenue, Braamfontein, Johannesburg, Gauteng, 2001, South Africa

# Correspondent author, E-mail: ipolikarpov@ifsc.usp.br

Supplementary Material & Methods

1. Bioinformatic analysis and target selection

Predicted polysaccharide utilization loci (PULs) containing enzymes from *Capnocytophaga ochracea* strain DSM 7271 (CoGH66, GenBank ID: WP_015782154.1) and *Prevotella melaninogenica* strain DSM 7089 (PmGH87, GenBank ID: WP_013264413.1) were sourced using the (CAZy: PULDB; http://www.cazy.org/PULDB/) Tool.The relevant PUL (PUL 9) for *C. ochracea* DSM 7271 was directly retrieved from the database. In contrast, due to the absence of *P. melaninogenica* DSM 7089 in the PULdb, *P. melaninogenica* strain D18, which contains predicted PUL 5, was selected for analysis, and the homologous proteins in *P. melaninogenica* DSM 7089 were identified using protein BLAST (see supplementary materials Table 1).

Signal peptide predictions were conducted using SignalP version 6.0 [1] and subcellular localization predictions were performed with DeepLoc-Pro [2], both accessed via the DTU bioinformatics platform. Phylogenetic analysis and sequence alignment were performed with CLUSTALW and ESPript3 respectively.

Nucleotide sequences coding for all glycoside hydrolases present in the *Capnocytophaga ochracea* (DSM 7271; NCBI:txid521097) PUL may be accessed via the NCBI database with the following accession numbers: (CoGH66; GenBank ID: WP_015782154.1; CoGH97) (GenBank ID: WP_015782153.1)

and Prevotella melaninogenica (DSM 7089/ATCC 25845, NCBI:txid553174) PUL (pPmPUL): (PmGH87; GenBank ID: WP_013264413.1), (PmGH99; GenBank ID: WP_013264030.1), (PmGH97; GenBank ID: WP_013264838.1)

The *Capnocytophaga ochracea* putative dextran utilization locus is made up of a SusR like Transcriptional regulator, a GH family 97 enzyme (CoGH97; Fig S10) and a GH family 66 enzyme (CoGH66), two cell Surface glycan binding proteins and a TonB-dependant transporter, (See Fig.1). CoGH97, featuring a type I signal peptide, was predicted to be localised to the periplasmic space with high confidence (0.9779), whilst CoGH66 and the two glycan binding proteins contained lipoprotein signal peptide type II, indicating likely transport to the outer membrane; CoGH66 was predicted to be localised at the outer membrane or extracellular space with low confidences, (0.5958) and (0.2796), respectively.

Additionally, The *P. melaninogenica* putative α-(1→3)-glucan utilisation locus contained a SusR like transcriptional regulator, GH family 87 (PmGH87) enzyme, two proteins of unknown function—a short and a conserved hypothetical protein, respectively (Fig. S11)—a TonB-dependent transporter, single SusD like glycan binding protein, and enzymes from GH families 71/99 (henceforth PmGH99) and 97 (PmGH97). (Fig. 1). PmGH87 and PmGH99 featured type II Lipoprotein signal peptides and were localised to the extracellular membrane with high (0.9445) and low (0.4518) confidences, respectively. The two hypothetical proteins were localised to the outer membrane and periplasmic spaces, both with low confidence (0. 5792) and (0.6407), respectively. Whilst the GH97 featured a type 1 signal peptide and was confined to the periplasmic with high confidence (0.9764

2 Enzyme source, cloning and purification:

Largely following the procedure of (Camilo and Polikarpov 2014). [3] In brief: Enzyme coding genes were selected and amplified from genomic DNA via PCR with Phusion® High-fidelity DNA polymerase (New England Biolabs) and primers designed with LIC sites complementary to the pETTRXA-1a/LIC vector. After annealing, the plasmids were

transformed into Escherichia coli competent cells by the heat shock method, first in the DH5-α strain for plasmid amplification and storage, followed by Rosetta(DE3) (CoGH66, CoGH97, PmGH97, PmGH99) or ArcticExpress™(DE3) (PmGH87) expression systems. After each transformation, the cells were mixed with a sterile glycerol solution and frozen at -80°C until further use. After glycerinated stocks of transformed cells have been prepared; Transformed cells expressing recombinant PmGH87 and CoGH66 were sourced from (A. Cortez). [4]

Day 1: prepare pre-inoculum, inoculate 10 µL of glycerinated stock into 10 ml LB media with 50 µg/ml kanamycin (Arctic) or kanamycin + 34 µg/ml chloramphenicol (Rosetta), (5ul stock solution each), and allow to grow overnight ~16h at 37C with shaking; Prepare 1L of LB media in 2L Erlen Myer flask and sterilize via autoclave.

Day 2: in a 2L Erlen Meyer flask inoculate 10ml pre-inoculum in 1L LB media (supplemented with the appropriate antibiotics (1ml each stock solution)), incubate approximately 4H at 37°C with shaking (growth phase) until OD600 reaches ~0.6 or the solution turns visibly opaque; thereafter, add IPTG to a final concentration of 1 mM (1ml stock solution) and allow to incubate at target temperature (23°C for Rosetta and 16°C for Arctic) and 200 rpm for 16h.

Day 3: Pellet cells by Centrifugation for 20 min at (10,000g; 4°C), remove supernatant and resuspend pellet in 50ml elution base buffer (PH 7.5; final concentrations: 50 mM Tris, 200 mM NaCl, 1 mM phenylmethanesulfonyl fluoride (PMSF)) at 4°C with shaking until resuspended, freeze at -80°C overnight.

Day 4: The resuspended cells were disrupted via 8 min of sonication on ice bath (30s on 30s off) using a 550 Sonic Dismembrator Sonifier (Fisher Scientific, Hampton, USA) at 40% duty cycle. The lysate was clarified by centrifugation at 15000 x g for 1h at 4°C to remove

cell debris, and the supernatant was collected and used for enzyme purification. Each supernatant was applied to 50ml Falcon centrifuge tube, each containing 5ml of Ni-NTA Superflow resin (Qiagen, Hilden, Germany), previously equilibrated with elution base buffer, and allowed to bind for 30m on orbital rotor. After which, washing and elution steps were carried out by successive fractions of imidazole (0mM, 30mM and 300mM) in 15ml base buffer, by centrifugation at 4700g for 20 min, after each cycle the supernatant is carefully collected. The eluted fractions were analysed by SDS-PAGE to identify fractions with the highest concentration of target protein used for subsequent experimental steps. Imidazole concentration of fractions was reduced to less than 10 mM by one or two successive steps of concentrating 10x in either a 50 kDa, or 10 kDa (PmGH99, PmGH97 and CoGH97), molecular cut-off concentrator and re-dilution with Base lysis buffer.

Day 5: The resulting sample were incubated with recombinant TEV protease (5:1 mass ratio) at 4°C for 54 h for 6xHis-thioredoxin tag removal from the target enzyme. Finally, a second $Ni^{2}+$ affinity chromatographic step was used for separation of tag-free enzymes from contaminants and TEV protease.

The purity of the protein sample obtained was confirmed by sodium dodecyl sulphate polyacrylamide gel electrophoresis (SDS-PAGE) under denaturing conditions. The purified enzyme was further concentrated, and the concentration was measured using Nanodrop 1000 spectrophotometer (Thermo Scientific, Waltham, EUA), at 280 nm and applying the calculated extinction coefficients. Unless specified otherwise enzymes were stored at a concentration of 2 mg/ml in storage buffer (PH 7.5; final concentrations: 50 mM Tris, 200 mM NaCl)

The CoGH66 used in biofilm experiments was a generous gift from Adelita Santiago.

Lyophilized His–Trx-tagged CoGH66 was reconstituted in mili-Q water, and the buffer was exchanged for enzyme storage buffer as described. To confirm comparable activity and validate the experimental approach, the specific activities of lyophilized His–Trx-tagged and non-lyophilized, non-tagged proteins were determined as 53.34 ± 1.498 and 60.35 ± 9.396 $\mu mol \cdot min^{-1} \cdot mg^{-1}$, respectively (see section 5). This supports the use of lyophilized His–Trx-tagged CoGH66 in subsequent biofilm experiments.

3 Biofilm forming Bacterial cultures

A brain-heart infusion (BHI) agar plate was coated with 10 µL of glycerol stock of Streptococcus mutans UA159 using a sterile cell spreader, until dry, to ensure uniform distribution of cells. The plate was incubated for 42 h at (37°C; 10% CO_2) in an anaerobic chamber to develop visible colonies.

To prepare the preinocula, a sterile inoculating loop was used to scrape a portion of bacterial growth from the surface of the BHI plate, under sterile conditions. The scraped biomass was transferred into a 50 ml falcon tube containing 10 ml of tryptone-yeast extract broth (TYEB) (2.5% tryptone, 1.5% Yeast extract w/v); Supplemented with glucose (1% w/v). The falcon tube was incubated overnight at (37°C; 10% CO_2) in an anaerobic chamber. Prior to biofilm assays, overnight cultures were mixed thoroughly by gentle inversion and standardized spectrophotometrically to an optical density of 1.0 ± 0.05.

To seed the biofilm growth media 200 µL of the adjusted preinoculum was added to 19.8 ml of TYEB supplemented with 1% sucrose (final concentration) to achieve a 1:100 dilution. The mixture was gently inverted to ensure even distribution of cells and grown in standard 24/96-well microtiter plates for 24 h at (37°C; 10% CO_2) in an anaerobic chamber. For 96-

well plates, 200 µL of the diluted culture was added to each well of a sterile, flat-bottomed 96-well plate; for 24-well plates, 2 ml of culture was used per well.

After the 24-hour incubation, the biofilm growth media was carefully removed from the 96-well plate. Each well was gently washed twice with 200 µL of saline solution (0.9% NaCl) to remove any residual media and non-adherent cells.

4 biofilm substrate and glucan preparation

Adapted from the protocol as described by (Cortez et al.), [4] Since commercial substrates for mutanases are not available, Lyophilized S. mutans bacterial pellets (whole biofilm; WBF) were prepared. In brief, S. *mutans* preinoculum was prepared as described in the previous section and used for the bacterial pellet preparation. 5 ml of this stock culture was transferred to 500 ml of a TYEB supplemented with 1% (29.2 mM) of sucrose and divided into 50 ml falcon tubes. The culture was incubated for 24 h at (37°C, 10% CO2). After growth, the bacterial cells and the biofilm matrix were harvested by centrifugation for 10 min at 10,000 g and 4°C. The pellets were sequentially solubilized by sonification (Digital Sonifier Unit, model S-150D; Branson Ultrasonics Corp., Danbury, CT, USA) in 50 ml of mili-Q water, (to remove leftovers of the culture broth), 0.01 M EDTA (to remove divalent ions), and mili-Q water again. The final pellet was freeze-dried and kept in the refrigerator until being used as substrate for enzyme characterization.

Water-soluble glucan (WSG) was extracted from WBF by sonication in Milli-Q water using a 550 Sonic Dismembrator Sonifier (Fisher Scientific, Hampton, USA) at 40% duty cycle. The suspension was centrifuged (10 min, 10,000 × g, 4°C) and the supernatant collected. WSG was precipitated by addition of three volumes of cold 92% ethanol and recovered by

centrifugation. The pellet was resuspended in Milli-Q water and re-precipitated as a washing step, followed by sequential washing with 0.01 M EDTA and Milli-Q water under identical precipitation and centrifugation conditions.

Water-insoluble glucan (WIG) was extracted from the remaining pellet by incubation in 1 M NaOH for 2 h at room temperature on an orbital rotor. The alkaline extract (supernatant) was separated from residual pellet by centrifugation (10 min, 10,000 g, 4°C) and neutralized with HCl prior to precipitation with three volumes of 92% ethanol as described above. The resulting precipitate was separated via centrifugation (10 min, 10,000 g, 4°C) to recover the pellet which was washed sequentially with Milli-Q water, 0.01 M EDTA, and Milli-Q water. Washing steps consisted of resuspension via sonication, followed by centrifugation (10 min, 10,000 g, 4°C) to recover the pellet.

Finaly WSG and WIG preparations were resuspended in 5 ml Milli-Q water (WIG samples were sonicated to facilitate resuspension) and lyophilized.

5 Enzymatic assays

Enzymatic activities were determined colorimetrically by quantifying the amount of product released using standard curves. Unless stated otherwise, enzymatic activity was determined using the dinitrosalicylic acid (DNS) method, which quantifies reducing end-groups of saccharides [5]. For reactions of PmGH97 with the synthetic substrate 4-Nitrophenyl-α-D-glucopyranoside (pNP-α-Glc), the reaction was quenched by addition of 1:1 (v:v) 1M Na2CO3; enzymatic activity was quantified directly by measuring the release of p-nitrophenol in solution via absorbance at 405 nm. Regarding the DNS method, the reactions were stopped by mixing 1:1 (v:v) with DNS reagent and boiling for 5 m to develop colour before absorbance was measured at 540 nm. [5]

Initial substrate screening was performed against polysaccharide substrates (soluble starch, lyophilized *S. mutans* biofilm and commercial *Leuconostoc* dextran). Reactions were conducted in 96-well PCR plates, with each 50 µL reaction containing 1 mg/ml enzyme and 1% (w/v) substrate (bacterial pellet, dextranase-treated pellet, commercial dextran, or soluble starch) in 50 mM MES buffer (pH 6.0), incubated at 40°C for either 10 m or 24 h.

To determine the pH profile of PmGH97 and PmGH99, reactions were performed in citrate phosphate buffer (PH ~2-8). For PmGH97 the reaction mixture, consisting of 0.1 mg/ml Enzyme and 10 mM PNP-alpha-Glc, was incubated for 10 m at 40°C before quantification as previously described. For PmGH99 the reaction mixture consisted of 1 mg/ml Enzyme and 1% Lyophilized S. mutans biofilm and was incubated for 1 h at 40°C before quantification via DNS method.

The activity of pPmPUL enzymes (PmGH87, PmGH97 and PmGH99) was assessed against various substrates prepared from *S. mutans* biofilm (WSG, WIG and WBF) by quantifying the amount of glucose equivalents released as a function of time (10 – 60 m). Wells contained glucan or WBF solutions (at final concentrations of 1% (w/v) in MES buffer (50 mM, pH 6.0, 150 mM NaCl)), and enzymes or control (appropriate volume of enzyme storage buffer). Reactions were performed in parallel by incubating at 40°C with agitation (1000 rpm) for 1h. Samples were withdrawn at time points 10, 30 and 60 min and were immediately mixed with DNS solution to stop the reactions and stored at 4°C until measurement. All samples were applied to a microtiter plate and boiled in parallel to develop colour before quantifying reducing sugars (as described above). Noting evaporation in isolated wells at the edge of the plate, which corresponded to 60 min of reaction time and 10 min of reaction with WSG, data corresponding to these conditions were discarded.

To assess potential activity of PmGH99 against yeast mannans, enzymatic assays were performed using whole cells of commercially available Saccharomyces cerevisiae as a substrate. Reaction mixtures contained PmGH99 at a final concentration of 1 mg·ml^{-1} and whole yeast cells at concentrations ranging from 0.1–10% (w/v) suspended in MES buffer (pH 6.0; final concentration 50 mM) supplemented with 150 mM NaCl. Reactions were incubated at 40 °C with agitation (1000 rpm) in a thermomixer for durations ranging from 10 min to 24 h. Afterwards reactions were stopped and quantified via DNS method.

To quantify activity of CoGH66 10 µg/ml of enzyme was incubated with commercial dextran (1% w/v) for 10 m at 50 °C, in MES buffer pH 5.5, before measuring reducing sugar using the dns method.

6 Biofilm formation and degradation assays

Adapted from. [6, 7] Biofilm degradation was quantified using crystal violet staining as follows. *S. mutans* biofilms were formed for 24 h in 96-well plates as previously described. Following biofilm formation, non-adherent cells and culture media were removed by inverting the plates and firmly tapping against paper towels and the biofilms on the bottom of the wells were washed twice with 200 ul saline solution which was added by pipetting slowly and gently against the walls of the well, careful not to disturb the biofilm.

For the enzymatic treatments, 200 ul of either enzyme solutions in MES buffer (PH 6, final concentration 50mM and 150mM NaCl) or experimental controls, which consisted of only MES buffer and the same volume of enzyme storage buffer (50 mM Tris PH 7.5, 200 mM NaCl) as the corresponding reaction, was added to the wells and incubated at 37°C for 4 h

After the enzymatic treatment, the remaining biofilms were once again washed twice with 200 ul saline solution as previously described. To stain the biofilms, 200 µL of 0.5% crystal violet was added to each well, and allowed to incubate for 15 m, followed by two additional washes with saline. The biofilms were then treated with 200 µL of 30% acetic acid for 15 minutes to recover the dye. The absorbance of the resulting solution was measured in a microplate reader at 570 nm to quantify the remaining biomass (%) in comparison to the controls.

Enzymatic treatments involved varying concentrations of PUL enzymes (PmGH87, PmGH97 and PmGH99) and CoGH66 applied individually, in binary combinations (total dose fixed at 1 mg/ml) of either PmGH87 and PmGH97 or PmGH87 and CoGH66 (positive control), or ternary combinations of PUL enzymes (total dose fixed at 1 mg/ml).

7 Confocal laser scanning microscopy (CLSM):

Imaging of endogenous fluorescence was performed using a Zeiss LSM 780 fluorescence confocal microscope equipped with an argon and HeNe laser as a source for excitation experiments with a Plan-Neofluar objective lens (10X, numerical aperture 0.3).

Biofilms were prepared in 24-well plates as previously described. All experiments were performed in MES buffer (PH 6, final concentration 50mM and 150mM NaCl); at 37°C for 2 h which was experimentally validated (see Fig. S6) Experimental controls consisted of only MES buffer and the same volume of enzyme storage buffer (50 mM Tris PH 7.5, 200 mM NaCl) as the corresponding reaction; all biofilms were inoculated from cultures prepared from the same colony and standardized in a spectrophotometer to an OD600 of 1.0 ± 0.05 and

grown for 24 h in standard 24-well microtiter plates prior to application of enzymatic treatments.

Supplementary tables

Table S1. Protein Blast results for Prevotella melaninogenica D18 against DSM 7089

Protein Name	% Coverage	% Identity
PmGH87	100	98.55
PmGH99	97	98.54
PmGH97	100	97.33

Table S2. Physicochemical properties of glycoside hydrolases identified in the *C. ochracea* and *P. melaninogenica* PUL, including molecular mass, isoelectric point (pI), and calculated extinction coefficients.

Protein Alias	NCBI code	Molecular Mass (kDa) Without tag	Calculated PI	Calculated extinction coefficient (E/1000)
CoGH66	WP_015782154.1	63.52	6.25	122.00
CoGH97	WP_015782153.1	80.906	6.47	147.85
PmGH87	WP_013264413.1	103.23	5.64	165.18
PmGH97	WP_013264838.1	73.479	6.45	145.44
PmGH99	WP_013264030.1	44.159	6.19	95.94

Table S3. Primers used in cloning of novel PUL GHs

Protein Alias	Forward primer (sequence 5’-3’)	Reverse primer (sequence 5’-3’)
CoGH97	CAGGGCGCCATGCAGGAGTATTTTCGCCTAAC	GACCCGACGCGGTTACTATCGTTTTTTTAATCCTTTGG
PmGH97	CAGGGCGCCATGAAGCAGGAAGCTACGGTGAG	GACCCGACGCGGTTATTACTTCAAACAAACCAACTGAA
PmGH99	CAGGGCGCCATGTGTGCAGACAGCTATGAGGG	GACCCGACGCGGTTATTAATTCTTACCATTAACAGCATC

Table S4. Calculated kinetic parameters for PmGH87 and CoGH66. CoGH66 kinetics were well described by the Michaelis–Menten model, with relative standard errors (RSE) below 10%. In contrast, RSE for PmGH87 exceeded 30%, reflecting poor constraint of the fitted parameters. PmGH87 did not reach saturation within the tested substrate range, limiting reliable estimation of kinetic parameters. The low maximum activity observed—which approached the detection limits of the assay—together with an apparent reduction in activity at higher substrate concentrations—possibly due to the insoluble and/or impure nature of the substrate—restricted the usable concentration range and likely contributed to the inability to achieve saturation under the conditions employed.

**Enzyme**	**Substrate**	**Vmax ±stdv (U/mg)**	**RSE (%) in Vmax**	**Km ±stdv**	**RSE (%) in Km**
CoGH66	Dextran	57.667 ± 1.620	2.81	0.583 ± 0.045 **(mM)**	7.72

PmGH87	WBF	0.609 ± 0.104 **b**	17.08	7.624 ± 2.339 **(mg/ml) b**	30.68

b = reaction did not reach saturation

Supplementary Figures:

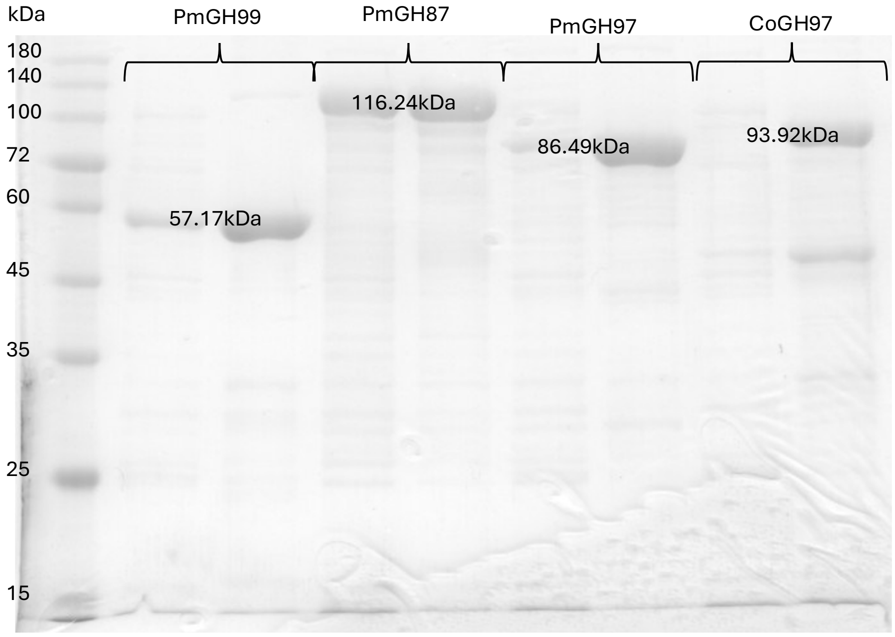

Supplementary Figure S1. SDS-PAGE (12% acrylamide) of GHs (with tag) used in initial screening experiments; lane 1 BlueClassic Prestained Protein Marker (10-180kDa)

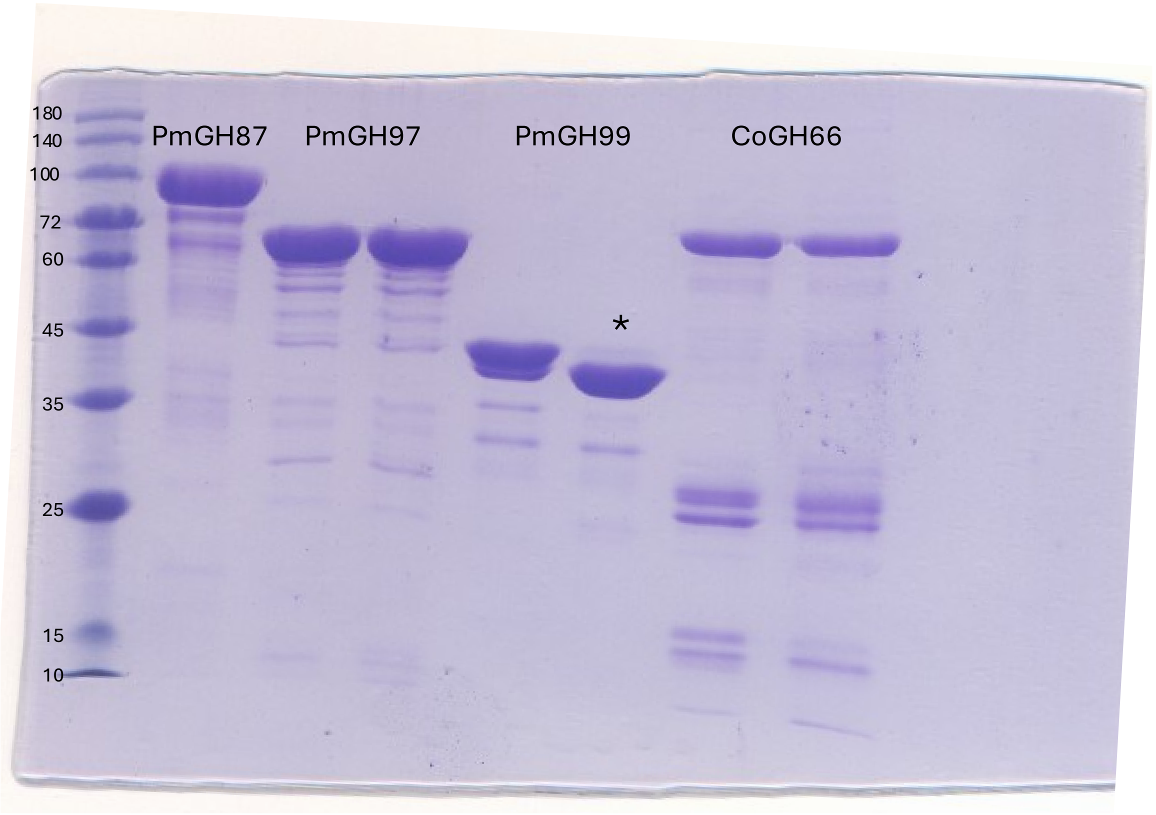

Supplementary Figure S2. SDS-PAGE (12% acrylamide) of GHs (without tag) used in biofilm experiments; lane 1 BlueClassic Prestained Protein Marker (10-180kDa)

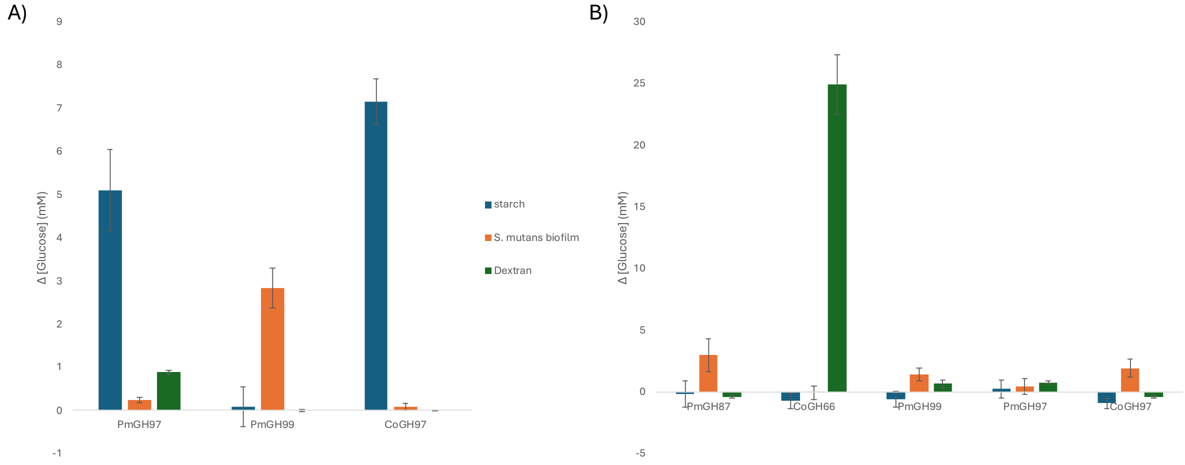

Supplementary Figure S3. (A) Substrate screening of novel recombinant enzymes (PmGH97, PmGH99/71, and CoGH97) against soluble starch, S. mutans pellet, and commercial dextran. Results indicate an increase in reducing sugars compared to substrates alone Reactions were performed with 1 mg/ml enzymes and 1% (w/v) substrates, incubated

for 24 h at 40°C, pH 5.5. **(B)** Substrate screening of all recombinant PUL enzymes (PmGH87, CoGH66, PmGH99/71, PmGH97, and CoGH97) against soluble starch, S. mutans pellet, and commercial dextran. Results indicate an increase in reducing sugars compared to substrates alone (Δ Glucose concentration (mM)) detected via the DNS method (Section 3.5). Reactions were performed with 1 mg/ml enzymes and 1% (w/v) substrates, incubated for 10 m at 40°C. pH 5.5.

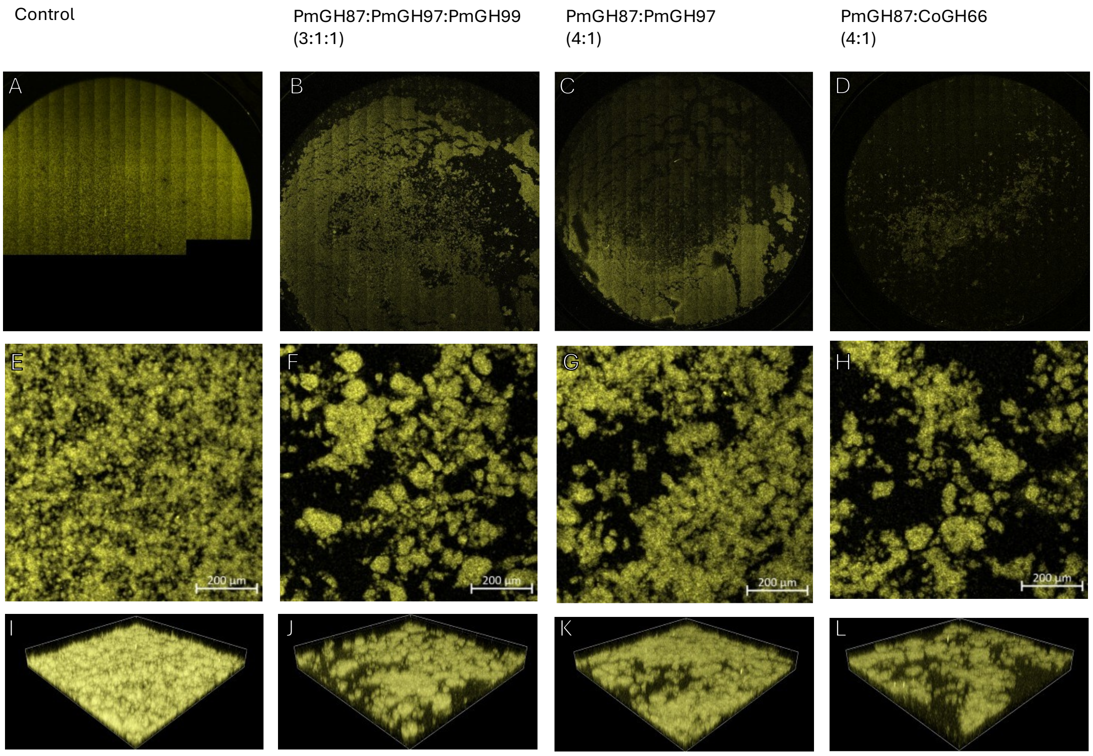

Supplementary Figure S4. CLSM images of *S. mutans* biofilms (10× magnification) following experimental treatments. (A–H) 2D slices: (A–D) reconstructed views of whole wells showing overall biofilm architecture; (E–H) selected regions highlighting finer structural details. (I–L) 3D reconstructions of corresponding regions. (A, E, I) control; (B, F, J) best-performing ternary combination (3:1:1 PmGH87:PmGH97:PmGH99); (C, G, K) binary mixture (4:1 PmGH87:PmGH97); (D, H, L) positive control (4:1 PmGH87:CoGH66). All treatments were applied at a fixed total enzyme concentration of 1 mg/ml. Experiments

were performed at 37 °C for 2 h in MES buffer (pH 6.0, 50 mM) supplemented with 150 mM NaCl.

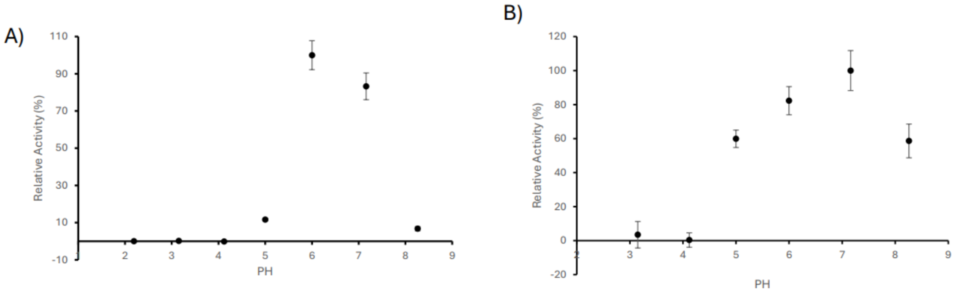

Supplementary Figure S5. PH profile of PmGH97 (A) and PmGH99 (B) – reactions were performed in citrate phosphate buffer (PH ~2-8). For PmGH97 the reaction mixture consisting of 0.1 mg/ml Enzyme, 10 mM PNP-alpha-Glc was incubated for 10 m at 40C and quenched 1:1 (v:v) with1M Na2CO3 before measuring absorbance at 405 nm. For PmGH99 the reaction mixture consisting of 1 mg/ml Enzyme and 1% Lyophilized S. mutans biofilm was incubated for 1 h at 40 C and stopped by mixing 1:1 (v:v) with DNS reagent and boiling for 5 m to develop colour before absorbance was measured at 540 nm.

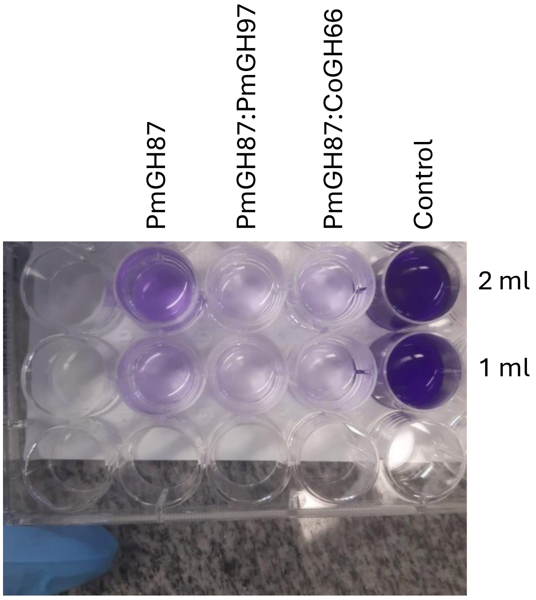

Supplementary Figure S6. Results of screening test for experimental conditions used in CLSM experiment; crystal violet assays of remaining biofilm after treatments (4 h) with enzyme mixtures; applied to biofilms grown in either 1 ml or 2 ml of media in a 24-well microtiter plate. Noting high biofilm degradation, a shorter incubation period (2h) was chosen for CLSM experiment to obtain images of the biofilm.

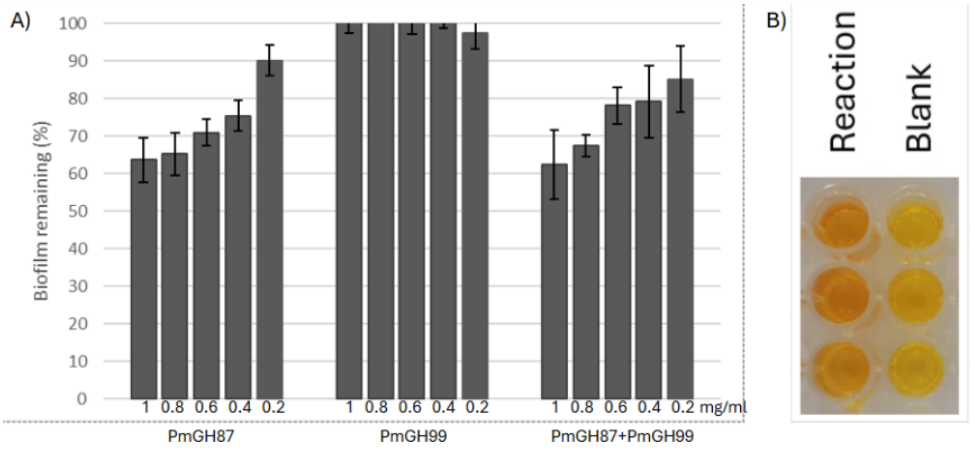

Supplementary Figure S7. (A) Results of biofilm degradation experiments of PmGH87 and PmGH99 in microtiter plate. For the mixed enzyme assays enzymes were applied with a ratio of 1:1 with total enzyme concentration indicated. All experiments performed at 36°C in MES

buffer (PH 6, final concentration 50mM and 150mM NaCl) at a final volume of 200 µL per well; Experimental controls consisted of only MES buffer and the same volume of enzyme storage buffer (50 mM Tris pH 7.5, 200 mM NaCl) as the corresponding reaction. (B) Results of enzymatic assay of PmGH99 against lyophilized S. mutans biofilm. Reaction conditions: 1% WBF, 1 mg/ml enzyme, performed in same buffer as biofilm degradation experiment (PH 6, final concentration 50mM and 150mM NaCl) at 40°C for 1 h.

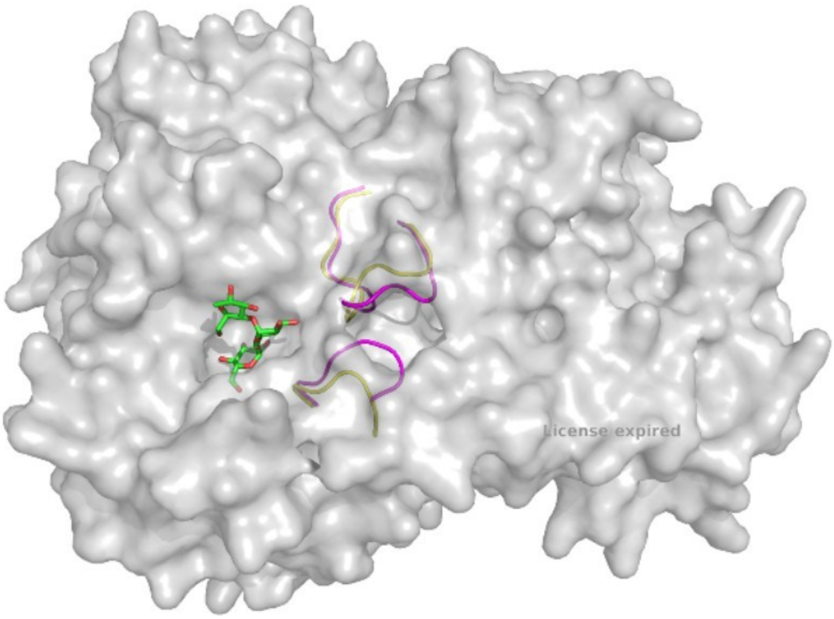

Supplementary Figure S8. Surface of PmGH97 (grey) aligned with PspAG97A complexed with panose (green). Differences in the loops in PmGH97 (coloured yellow) corresponding to Y408-N414 and I164-Y176 in PspAG97A (coloured magenta), reveals an additional pocket of unknown function in the PmGH97 predicted structure not observed in other members.

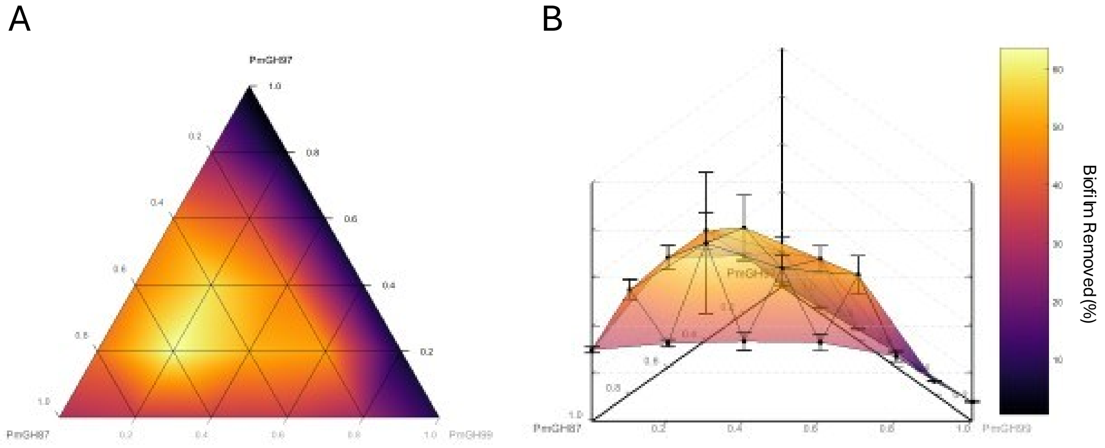

Supplementary Figure S9. Biofilm removal effect of ternary combinations of enzymes, with total enzymatic dose fixed at 1 (mg/ml) represented as ternary contour plots (A) and with rotated view showing the surface response mean ± standard deviation (n = 4) (B); All experiments performed at 37°C for 4 h in MES buffer (PH 6, final concentration 50mM and 150mM NaCl); Experimental controls consisted of only MES buffer and the same volume of enzyme storage buffer (50 mM Tris PH 7.5, 200 mM NaCl) as the corresponding reaction; Experiments were performed on separate days; all biofilms were inoculated from cultures prepared from the same colony and standardized in a spectrophotometer to an OD600 of 1.0 ± 0.05 and grown for 24 h before application of enzymatic treatments.

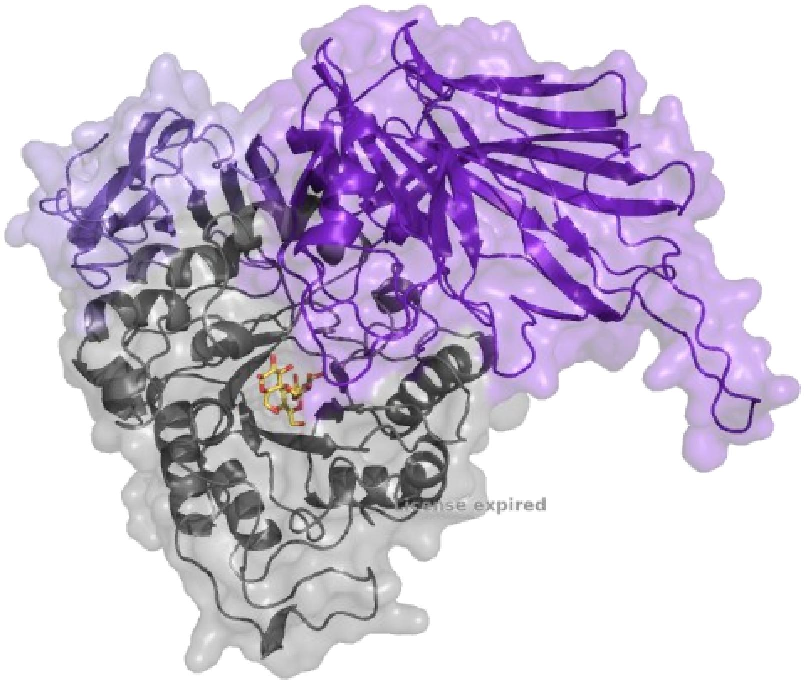

Supplementary Figure S10. Cartoon representation of the predicted structure of CoGH97, superimposed with panose (yellow) in the catalytic cleft; N-terminal domain (purple), Catalytic domain (grey), and C-terminal domain (blue-violet).

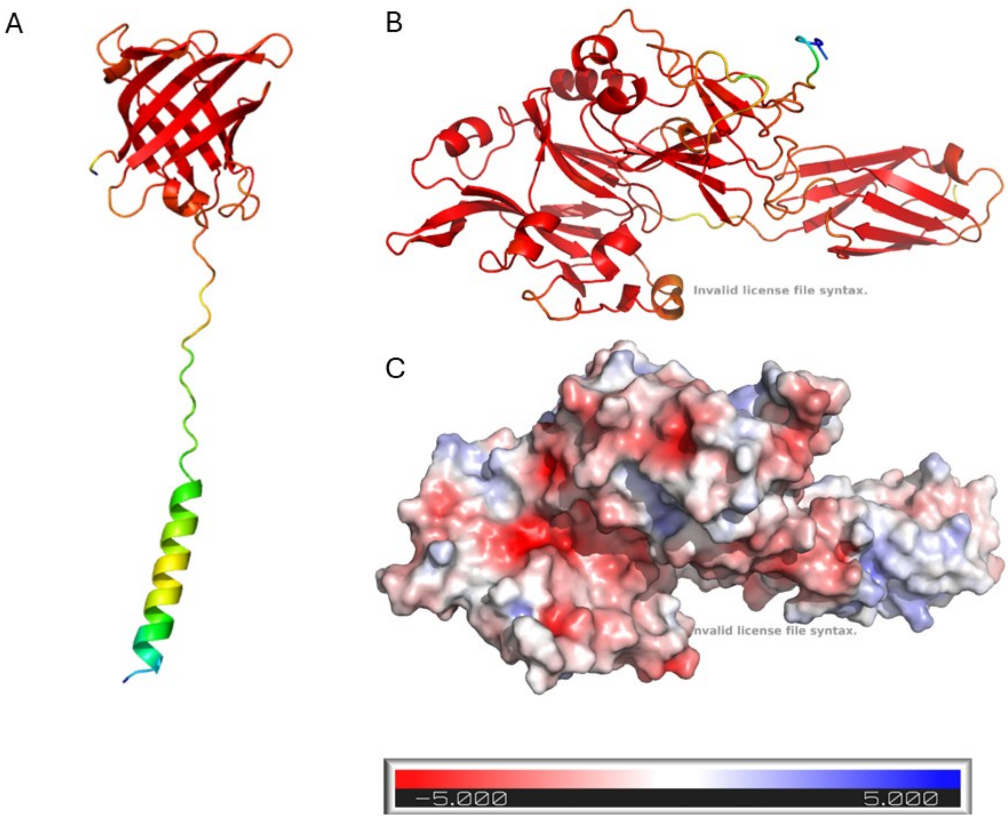

Supplementary Figure S11. Cartoon representations of predicted structures of the pPmPUL short (A) and conserved (B) hypothetical proteins, respectively, of unknown function. chains colored by per-residue confidence (pLDDT) with red being the most confident. (C) Surface representation of the predicted hypothetical conserved protein colored by charge.

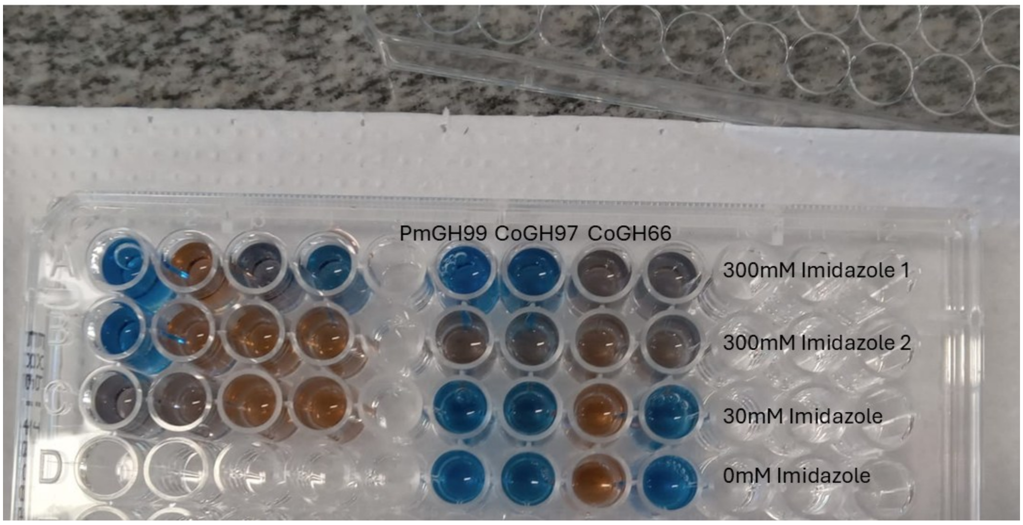

Supplementary Figure S12. Representative protein purification run showing elution fractions collected during affinity chromatography with an imidazole gradient. Protein concentration was estimated using Bradford reagent, where increasing blue colour intensity

corresponds to higher protein concentration. Elution fractions at low concentrations of imidazole correspond to protein impurities lacking the poly-His-tag and higher concentrations correspond to the partially purified GHs.

References:

[1] "SignalP 5.0 improves signal peptide predictions using deep neural networks | Nature Biotechnology." Accessed: Apr. 06, 2026. [Online]. Available: https://www.nature.com/articles/s41587-019-0036-z

[2] "Predicting the subcellular location of prokaryotic proteins with DeepLocPro | Bioinformatics | Oxford Academic." Accessed: Apr. 06, 2026. [Online]. Available: https://academic.oup.com/bioinformatics/article/40/12/btae677/7900293?guestAccessKey=

[3] C. M. Camilo and I. Polikarpov, "High-throughput cloning, expression and purification of glycoside hydrolases using Ligation-Independent Cloning (LIC)," *Protein Expr. Purif.*, vol. 99, pp. 35–42, Jul. 2014, doi: 10.1016/j.pep.2014.03.008.

[4] "Recombinant Prevotella melaninogenica α-1,3 glucanase and Capnocytophaga ochracea α-1,6 glucanase as enzymatic tools for in vitro degradation of S. mutans biofilms | World Journal of Microbiology and Biotechnology | Springer Nature Link." Accessed: Apr. 06, 2026. [Online]. Available: https://link.springer.com/article/10.1007/s11274-023-03804-z

[5] G. L. Miller, "Use of Dinitrosalicylic Acid Reagent for Determination of Reducing Sugar," *Anal. Chem.*, vol. 31, no. 3, pp. 426–428, Mar. 1959, doi: 10.1021/ac60147a030.

[6] E. F. Haney, M. J. Trimble, and R. E. W. Hancock, "Microtiter plate assays to assess antibiofilm activity against bacteria," *Nat. Protoc.*, vol. 16, no. 5, pp. 2615–2632, May 2021, doi: 10.1038/s41596-021-00515-3.

[7] B. M. Coffey and G. G. Anderson, "Biofilm Formation in the 96-Well Microtiter Plate," in *Pseudomonas Methods and Protocols*, A. Filloux and J.-L. Ramos, Eds., New York, NY: Springer, 2014, pp. 631–641. doi: 10.1007/978-1-4939-0473-0_48.
